# Premature termination codon readthrough upregulates progranulin expression and improves lysosomal function in preclinical models of *GRN* deficiency

**DOI:** 10.1186/s13024-020-00369-5

**Published:** 2020-03-16

**Authors:** Jonathan Frew, Alireza Baradaran-Heravi, Aruna D. Balgi, Xiujuan Wu, Tyler D. Yan, Steve Arns, Fahimeh S. Shidmoossavee, Jason Tan, James B. Jaquith, Karen R. Jansen-West, Francis C. Lynn, Fen-Biao Gao, Leonard Petrucelli, Howard H. Feldman, Ian R. Mackenzie, Michel Roberge, Haakon B. Nygaard

**Affiliations:** 1 Division of Neurology, University of British Columbia, Vancouver, British Columbia Canada; 2 Department of Biochemistry and Molecular Biology, University of British Columbia, Vancouver, British Columbia Canada; 3 adMare BioInnovations, Vancouver, British Columbia Canada; 4 JAQJAM Consulting, Cobourg, Ontario Canada; 5 Department of Neuroscience, Mayo Clinic, Jacksonville, FL USA; 6 Department of Surgery, University of British Columbia, Faculty of Medicine, Vancouver, British Columbia Canada; 7 Department of Neurology, University of Massachusetts Medical School, Worcester, MA USA; 8 Department of Neurosciences, University of California, San Diego, San Diego, CA USA; 9 Department of Pathology and Laboratory Medicine, University of British Columbia, Vancouver, British Columbia Canada

**Keywords:** Progranulin, GRN, Frontotemporal lobar degeneration, Nonsense mutation, Premature termination codon, Readthrough, G418, Induced pluripotent stem cell, Neurons

## Abstract

**Background:**

Frontotemporal lobar degeneration (FTLD) is a devastating and progressive disorder, and a common cause of early onset dementia. Progranulin (PGRN) haploinsufficiency due to autosomal dominant mutations in the progranulin gene (*GRN*) is an important cause of FTLD (FTLD-*GRN*), and nearly a quarter of these genetic cases are due to a nonsense mutation. Premature termination codons (PTC) can be therapeutically targeted by compounds allowing readthrough, and aminoglycoside antibiotics are known to be potent PTC readthrough drugs. Restoring endogenous PGRN through PTC readthrough has not previously been explored as a therapeutic intervention in FTLD.

**Methods:**

We studied whether the aminoglycoside G418 could increase PGRN expression in HEK293 and human induced pluripotent stem cell (hiPSC)-derived neurons bearing the heterozygous S116X, R418X, and R493X pathogenic *GRN* nonsense mutations. We further tested a novel substituted phthalimide PTC readthrough enhancer in combination with G418 in our cellular models. We next generated a homozygous R493X knock-in hiPSC isogenic line (R493X^−/−^ KI), assessing whether combination treatment in hiPSC-derived neurons and astrocytes could increase PGRN and ameliorate lysosomal dysfunction relevant to FTLD-*GRN*. To provide in vivo proof-of-concept of our approach, we measured brain PGRN after intracerebroventricular administration of G418 in mice expressing the V5-tagged *GRN* nonsense mutation R493X.

**Results:**

The R418X and R493X mutant *GRN* cell lines responded to PTC readthrough with G418, and treatments increased PGRN levels in R493X^−/−^ KI hiPSC-derived neurons and astrocytes. Combining G418 with a PTC readthrough enhancer increased PGRN levels over G418 treatment alone in vitro. PGRN deficiency has been shown to impair lysosomal function, and the mature form of the lysosomal protease cathepsin D is overexpressed in R493X^−/−^ KI neurons. Increasing PGRN through G418-mediated PTC readthrough normalized this abnormal lysosomal phenotype in R493X^−/−^ KI neuronal cultures. A single intracerebroventricular injection of G418 induced *GRN* PTC readthrough in 6-week-old AAV-*GRN*-R493X-V5 mice.

**Conclusions:**

Taken together, our findings suggest that PTC readthrough may be a potential therapeutic strategy for FTLD caused by *GRN* nonsense mutations.

## Background

Autosomal dominant mutations in the progranulin gene (*GRN)* represent a major genetic cause of frontotemporal lobar degeneration (FTLD) accounting for 5–10% of all cases [1–3]. The vast majority of these cases are due to *GRN* nonsense mutations, deletions, or splice-site mutations, leading to progranulin (PGRN) haploinsufficiency. Since the discovery of PGRN haploinsufficiency as a major cause of FTLD, there has been an ongoing search for interventions to raise central nervous system progranulin as a therapeutic strategy. These strategies have thus far revolved around non-specific mechanisms derived from high throughput drug screens as well as more specific efforts targeting the PGRN signaling pathway or replacing the protein through adeno-associated virus (AAV) gene therapy [4–7]. While nearly a quarter of all *GRN* linked FTLD (FTLD-*GRN*) cases are due to a *GRN* nonsense mutation [1], to our knowledge suppression of endogenous nonsense mutations has not been pursued as a therapeutic strategy despite significant interest in this approach in other neurologic conditions [8–10]. Nonsense mutations change an amino acid codon to a termination codon (UGA, UAG, or UAA) resulting in the production of a truncated protein and mRNA destabilization [11]. Several compounds, including aminoglycoside antibiotics, can suppress nonsense mutations by enabling pairing of a near-cognate aminoacyl-tRNA at a PTC, allowing for the incorporation of an amino acid instead of termination, leading to translation of the full-length protein and increased nonsense mutant mRNA stability [11–13]. However, given the narrow therapeutic index of aminoglycosides, supplementary compounds that enhance their PTC readthrough activity may allow for lower drug dosing and thus better tolerability in humans. In a previous study, we discovered a novel class of aminoglycoside PTC readthrough enhancer compounds (CDX series) using a high-throughput screen for nonsense suppression in yeast [14]. We demonstrated that CDX5–1 enhanced aminoglycoside PTC readthrough by G418 in *TP53* nonsense mutant HDQ-P1 cancer cells [14]. In the present study, we aimed to investigate whether *GRN* nonsense mutations are susceptible to G418-mediated PTC readthrough in preclinical models of FTLD-*GRN*, including neurons and astrocytes from patient-derived induced pluripotent stem cells (iPSCs). We further tested whether treatment without or with a readthrough enhancer could reverse aberrant lysosomal phenotypes previously described in several preclinical models of FTLD-*GRN* as well as neuronal ceroid lipofuscinosis (NCL) due to progranulin deficiency [15–17]. Our findings provide proof-of-concept evidence that PTC readthrough is a promising avenue for therapeutic development for the treatment of FTLD-*GRN* patients bearing *GRN* nonsense mutations.

## Methods

### *GRN* expression vector mutagenesis

Using GeneArt mutagenesis service (Thermo Fisher Scientific) coding sequence of *GRN* was synthesized and cloned into pDONR221 Entry vector. Three base substitutions c.347C > A, c.1252C > T and c.1477C > T were engineered into *GRN* using a targeted PCR-based strategy to generate S116X (TAA), R418X (TGA) and R493X (TGA) nonsense mutations. Finally, to generate expression clones LR recombination reaction was used to recombine the mutated samples from the Entry vectors into the pcDNA-6.2/V5-DEST vector (Thermo Fisher Scientific). These C-terminal V5 tagged *GRN* expression constructs were used for HEK293 cell transfections.

### Transfection and generation of stable HEK293 cell lines

HEK293 cells were transiently transfected with pcDNA6.2/V5-DEST vector expressing C-terminally V5-tagged mutated *GRN* with nonsense mutations (S116X, R418X, R493X) using Lipofectamine 2000 (Thermo Fisher Scientific). Twenty-four hours after transfection, each sample was split into two wells and either left untreated or treated with G418 (100 μg/mL). After 72 h, cells were lysed and subjected to automated capillary electrophoresis western analysis (ProteinSimple WES). To generate stable cell lines, HEK293 cells were transfected with pcDNA-6.2/V5-DEST vectors expressing mutant *GRN* as described above and subjected to blasticidin selection (Thermo Fisher Scientific). Individual clones that were resistant to 15 μg/mL blasticidin were selected.

### Generation of hiPSCs and neuronal/astrocyte differentiation

Erythroid progenitor (EP) cells were isolated from peripheral blood obtained from a healthy control subject (WT) and a *GRN* R418X mutation carrier (R418X^+/−^) [18] using the erythroid progenitor reprogramming kit (STEMCELL Technologies). Expanded EPs were reprogrammed into hiPSCs with the Epi5™ episomal reprogramming kit (Invitrogen, Thermo Fisher Scientific) plasmids in Amaxa™ Human CD34^+^ Cell nucleofection buffer (Lonza) using the Amaxa™ Nucleofector II (Lonza) electroporation device according to the erythroid progenitor reprogramming kit manufacturer’s instructions. hiPSCs were cultured in a feeder-independent manner on matrigel (BD Biosciences) coated plates and fed daily with mTeSR1 medium (STEMCELL Technologies). Every 4–5 days (~ 80% confluence) cultures were passaged as aggregates using ReLeSR (STEMCELL Technologies) at ~ 1:5 split ratio. During the first 24 h post-plating, mTeSR1 medium was supplemented with 10 μM rho-associated protein kinase inhibitor (Y-27632, EMD Millipore). S116X^+/−^ hiPSCs were obtained from existing stock and previously described [19].

An isogenic hiPSC line homozygous for *GRN* R493X knock-in (R493X^−/−^ KI) was generated from WT using a combination of previously established CRISPR/Cas9 gene-editing protocols. Guide RNA (gRNA) sequences were designed according to the Optimized CRISPR Design online tool (http://crispr.mit.edu) to target a region for double-strand breakage slightly upstream of the *GRN* R493 codon. A mutagenizing single-stranded oligonucleotide (ssODN, Integrated DNA Technologies) was designed to knock-in the R493X mutation and silently introduce a HindIII restriction enzyme site. hiPSCs were co-transfected (Lipofectamine Stem Transfection Reagent, Invitrogen) with knock-in ssODN and Cas9/gRNA ribonucleoprotein complexes for 72 h. CRISPR edited hiPSCs were seeded at clonal density (25 cells / cm^2^). Once colonies were of adequate size and morphology, individual colonies were picked and plated for expansion. Genomic DNA was isolated using QuickExtract DNA Extraction Solution (Lucigen, Middleton, WI) and PCR products containing the R493X target site were digested with HindIII. Clones with positive digestion signal were selected for Sanger sequencing to confirm the clean introduction of the R493X KI mutation. Sanger sequencing was performed by the University of British Columbia Sequencing + Bioinformatics Consortium.

WT and FTLD-*GRN* mutant hiPSCs were differentiated into neuronal progenitor cells (NPCs) using the dual SMAD inhibition protocol [20]. These NPCs were frozen and thawed as needed onto matrigel coated plates in neural stem cell medium for week-long expansion. Expanded NPCs were then plated onto poly-L-ornithine / laminin (PLO/L) coated plates and further differentiated into cortical neurons using complete BrainPhys™ (STEMCELL Technologies) media system [21] supplemented with 1X CultureOne™ (Gibco, Thermo Fisher Scientific) for the first 2 weeks of neuron maturation. cAMP and ascorbic acid were withdrawn from BrainPhys™ media after 3 weeks (days in vitro from hiPSC stage, DIV 50) for continued neuronal maturation until DIV 80 to allow lysosomal phenotypes to develop. Astrocytes were differentiated from expanded cryopreserved NPCs according to the STEMDiff™ astrocyte differentiation and maturation kit (STEMCELL Technologies) protocol to produce DIV 60+ frozen stocks to be thawed and passaged as needed onto matrigel coated plates in STEMDiff™ astrocyte maturation medium.

### Drug administration

G418 sulphate powder was reconstituted in sterile PBS to 50 mg/mL and CDX series compounds in dimethyl sulfoxide (DMSO) to 50 mM. These stock solutions were stored at − 20 °C. Upon addition of CDX compounds to cell culture media, gentle vortexing was applied for 2 min to ensure complete solubilization. Vehicle solutions were prepared by adding corresponding volumes of PBS and DMSO. Recombinant PGRN (Adipogen, AG-40A-0068Y-C010) was reconstituted to 0.1 mg/mL in sterile water. Cathepsin L inhibitor (Z-Phe-Phe-FMK, Abcam, ab141386) 10 mM stock in DMSO was first diluted to 100 and 300 μM, prior to 1:10 dilution in cell culture medium. In vivo treatment solutions of vehicle (1.6% solutol), 16.6 mg/mL G418 (1.6% solutol), 0.5 mM CDX5–288 (1.6% solutol), and 16.6 mg/mL G418 + 0.5 mM CDX5–288 (1.6% solutol) were prepared in sterile saline. A 50 mM solution of CDX5–288 in DMSO was first diluted to 3 mM in 10% solutol (diluted in sterile saline) and further diluted 1:6 in treatment solutions.

### Chemical synthesis of CDX5–196 and CDX5–288

#### 6-((2,6-dichlorobenzyl)amino)isoindolin-1-one (CDX5–196)

A solution of 6-amino-2, 3-dihydro-1H-isoindol-1-one (825 mg, 1.0 eq), 2,6-dichlorobenzaldehyde (975 mg, 1.0 eq) and AcOH (0.32 mL, 1.0 eq) in dichloroethane (56 mL) was treated with NaBH(OAc)_3_ (1.54 g, 1.3 eq) and stirred at room temperature for 72 h. Additional aliquots of 2,6-dichlorobenzaldehyde (488 mg, 1.0 eq) and NaBH(OAc)_3_ (592 mg, 0.5 eq) were added and the reaction was stirred for a further 24 h. The reaction was then quenched with saturated aqueous NaHCO_3_ (50 mL) and the layers were separated. The aqueous layer was extracted with dichloromethane (2 × 25 mL). The organic layers were combined, dried over Na_2_SO_4_, filtered and concentrated to give the crude product. This solid was triturated with acetone. Filtration and thorough drying gave CDX5–196 (975 mg, 57% yield) as an off-white solid. ^1^H NMR (400 MHz, DMSO-*d*_6_) δ = 8.35 (s, 1H), 7.53 (d, *J* = 8.0 Hz, 2H), 7.39 (dd, *J* = 8.7, 7.4 Hz, 1H), 7.27 (d, *J* = 8.1 Hz, 1H), 6.98–6.92 (m, 2H), 6.04 (t, *J* = 5.0 Hz, 1H), 4.43 (d, *J* = 5.0 Hz, 2H), 4.21 (s, 2H). ^13^C NMR (101 MHz, DMSO) δ = 171.03, 149.07, 136.14, 134.46, 133.96, 132.10, 130.70, 129.13, 124.19, 117.37, 104.69, 44.74, 43.75.

#### 7-((2,6-dichlorobenzyl)amino)isoquinolin-1(2H)-one (CDX5–288)

To a mixture of 7-aminoisoquinolin-1(2H)-one (100 mg, 1.0 eq), 2,6-dichlorobenzaldehyde (82 mg, 1.0 eq) and acetic acid (35 μL, 0.1 eq) in dichloroethane (6 mL) was added NaBH(OAc)_3_ (145 mg, 1.1 eq). The reaction mixture was stirred at room temperature for 24 h. An additional portion of NaBH(OAc)_3_ (395 mg, 3.0 eq) was added and the reaction mixture was stirred for another 24 h. The reaction was quenched with water and the aqueous layer was extracted with dichloromethane. The combined organic layers were dried over Na_2_SO_4_, filtered and concentrated. Purification via preparative HPLC (acetonitrile:water with 0.1% trifluoroacetic acid) afforded CDX5–288 (55 mg, 28% yield) as a light brown solid. ^1^H NMR (400 MHz, DMSO-*d*_6_) δ = 10.94 (d, *J* = 5.3 Hz, 1H), 7.53 (d, *J* = 8.1 Hz, 2H), 7.44–7.37 (m, 2H), 7.35 (d, *J* = 2.5 Hz, 1H), 7.15 (dd, *J* = 8.6, 2.6 Hz, 1H), 6.87 (dd, *J* = 7.0, 5.5 Hz, 1H), 6.39 (d, *J* = 7.0 Hz, 1H), 6.23 (s, 1H), 4.47 (d, *J* = 4.8 Hz, 2H). ^13^C NMR (101 MHz, DMSO-*d*_6_) δ = 162.12, 147.81, 136.18, 134.30, 130.74, 129.11, 128.76, 127.96, 127.50, 124.43, 120.80, 105.38, 105.17, 43.52.

### Antibodies

The antibodies used in this study were NANOG (R&D Systems, AF1997, 1:500), OCT4 (STEMCELL Technologies, 60093, 1:1000), SOX1 (STEMCELL Technologies, 60095, 1:1000), SOX17 (R&D Systems, AF1924, 1:500), DESMIN (Invitrogen, PA5–16705, 1:500), MAP2 (Proteintech, 17490–1-AP, 1:500), TUJ1 (Neuromics, CH23005, 1:500), GFAP (STEMCELL Technologies, 60128, 1:500), TBR1 (Abcam, ab31940, 1:500), FOXG1 (Abcam, ab18259, 1:500), SYNAPSIN (EMD Millipore, 5747777, 1:500), VGLUT1 (Synaptic Systems, 135311, 1:500), GAD 65/67 (Sigma-Aldrich, G5163, 1:500), PGRN (R&D Systems, AF2420, 1:1000) (for immunofluorescence); PGRN (Sigma-Aldrich, HPA008763, 1:1000), CTSD (R&D Systems, AF1014, 1:1000), ACTIN (Novus Biologicals, NB600–532, 1:10,000) (for conventional western blot); V5 (Abcam, ab27671, 1:500), ACTIN (Novus Biologicals, NB600–532, 1:10,000) (for automated WES western blot) (Table S1).

### Brightfield microscopy

Live cultures were photographed with a digital camera mounted to a tissue culture microscope (40X obj. lens) throughout the differentiation process from hiPSCs to cortical neurons.

### Immunofluorescence microscopy

Cells were fixed with 4% paraformaldehyde (PFA) for 15 min and washed three times with Dulbecco phosphate-buffered saline (D-PBS, Gibco, Thermo Fisher Scientific). Cells were blocked and permeabilized with 10% donkey or goat serum (Sigma-Aldrich) in D-PBS containing 0.1% triton X-100 (Abcam) for 1 h at room temperature (RT). Primary antibodies were then diluted (see Antibodies Methods section or Table S1) in 10% donkey or goat serum in D-PBS and applied to cells overnight at 4 °C. all Alexa Fluor-tagged secondary antibodies (Invitrogen, Thermo Fisher Scientific) were used at a dilution of 1:500 at RT for 2 h. the coverslips were then mounted in DAPI mounting medium (Vector Laboratories). Images were captured with ZEN 2 software using a Zeiss 880 scanning laser confocal microscope. Image quantification was performed with NIH ImageJ. The % of neurons expressing specific markers were determined by applying uniform thresholds and converting images to binary using watershed segmentation tool and quantifying the number of individual DAPI+/neuronal marker+ cells with the analyze particles function. Cytotoxicity was assayed by staining fixed (as above) 96-well plate neuronal cultures with Hoechst dye (1.5 μg/mL, H3570, Invitrogen). These plates were imaged using a Cellomics ArrayScan™ plate scanner (Thermo Fisher Scientific) and the number of cells were automatically counted with acQuisition™ software (Thermo Fisher Scientific).

### Multielectrode array electrophysiology

WT and mutant hiPSC-derived NPCs (DIV 30) were co-cultured with hiPSC-derived WT astrocytes (DIV 60+) in 1:1 ratio and plated onto 48-well multielectrode array (MEA) plates (Axion BioSystems) coated with PLO/L. Cells were plated using drop seed method, where 15 μL of mixed cell suspension (60,000 cells in total) in a 10% dilution of matrigel in BrainPhys™ + 1X CultureOne™ media was pipetted onto the electrode array in the center of each well. The cultures were fed by partial media replacement every 3 days according to our neuronal differentiation protocol stated above. Spontaneous electrophysiological activity of the neuron-astrocyte co-cultures was recorded for 10 min at cortical neuron DIV 50 using the Axion biosystems maestro MEA at 37 °C and 5% CO_2_. Data analysis was performed using AxIs software (Axion BioSystems) to extract the number of spikes and bursts from the recording file. Quality criteria for the assays were defined as follows: an electrode having an average of more than 5 spikes/min and wells with less than 30% of the total electrodes active were considered inactive and excluded from the analysis.

### Conventional western blot

Cortical neuron and astrocyte supernatant were collected, and cell monolayers were rinsed with 1 mL ice-cold PBS. Cells were lysed in 50 μl lysis buffer (20 mM Tris–HCl pH 7.5, 150 mM NaCl, 1 mM EDTA, 1 mM EGTA, 1% (v/v) triton X-100, 2.5 mM sodium pyrophosphate, 1 mM β-glycerophosphate) supplemented with fresh 1 mM Na_3_VO_4_, and 1X complete protease inhibitor cocktail (Roche Molecular Biochemicals). Lysates and supernatants were pre-cleared by centrifugation at 14,000 rpm (19,500 x g) for 10 min at 4 °C. lysate protein concentration was quantitated using the Bradford assay. In brief, 10–30 μg protein from each boiled SDS lysate was separated on 4–15% gradient precast polyacrylamide gel (Bio-Rad Laboratories), electroblotted onto a nitrocellulose membrane and blocked for 1 h in 5% (w*/*v) non-fat milk. Membranes were incubated with primary antibodies overnight at 4 °C, washed three times with TBS + 0.1% (v/v) Tween-20 (TBS-T), incubated with HRP-conjugated secondary antibody, washed again 3X with TBS-T and incubated with enhanced chemiluminescence substrate (EMD Millipore). Films were developed, scanned, and analyzed using NIH ImageJ for densitometry analysis.

### ProteinSimple WES analysis

**Fig. 1:**
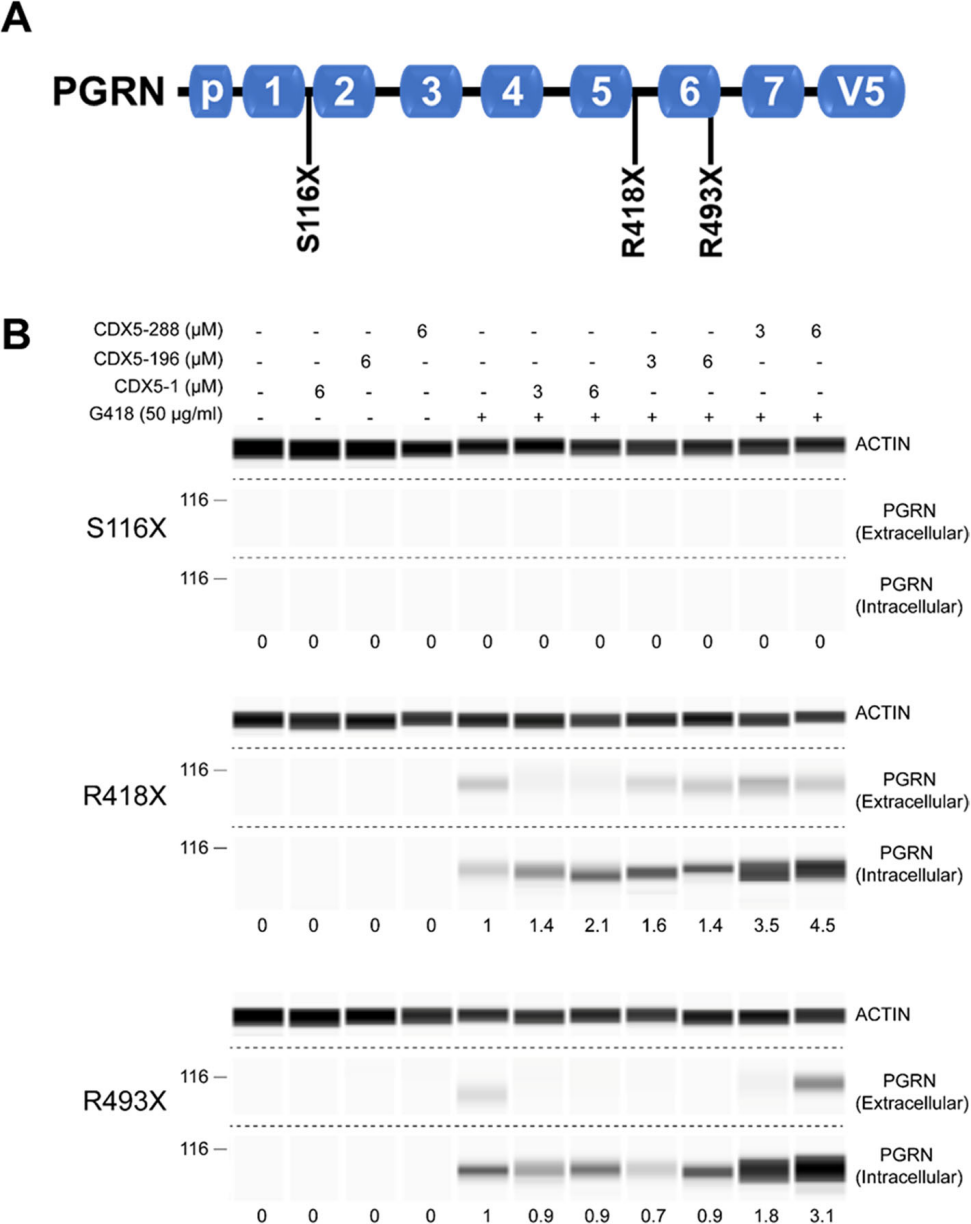
Induction of PTC readthrough by G418 and CDX5 enhancers in cells expressing GRN-V5. **a** Schematic of full-length PGRN highlighting the position of the S116X (UAA), R418X (UGA), and R493X (UGA) nonsense mutations in relation to the position of individual granulin peptides and the C-terminal V5 tag. **b** HEK293 cell lines stably expressing *GRN-V5* with the indicated nonsense mutations were treated with G418 and the indicated concentrations of CDX5–1, CDX5–196, and CDX5–288 for 72 h. Cell culture supernatants (extracellular) and cell lysates (intracellular) were subjected to automated capillary electrophoresis western analysis. Full-length PGRN was detected with a V5 antibody. Actin was measured in cell lysates as a loading control. The readthrough enhancement ratios are indicated under the lanes. The proportion loaded was 15–20 fold lower for the extracellular samples than for the intracellular samples

Automated capillary electrophoresis western analysis was carried out with manufacturer’s reagents according to the user manual (ProteinSimple WES). HEK293 cell lysates were prepared following the method used for neurons/astrocytes and AAV brain lysates were prepared according to brain tissue lysate preparation method section. Briefly, 5.6 μl of 1 mg/mL HEK293 cell/brain lysate was mixed with 1.4 μl fluorescent master mix and heated at 95 °C for 5 min. The samples, blocking reagent, wash buffer, primary antibody, secondary antibody, and chemiluminescent substrate were dispensed into the microplate provided by the manufacturer. The electrophoretic separation and immunodetection were performed automatically using the WES instrument default settings. PGRN was quantified using size-based detection with the integrated compass software (ProteinSimple). The full-length V5 (PGRN) peak intensities (area under the curve) were normalized to that of the actin peak, used as a loading control. In Fig. 1 and Fig. 8 electropherograms are represented as pseudo-blots, generated from the quantification of chemiluminescence by the compass software.

### Progranulin ELISA

Progranulin levels in neuronal and astrocyte whole cell lysates (as prepared for western blot) and concentrated supernatants (Amicon Ultra 0.5 mL, 50 kDa, 25X concentrated) were determined by ELISA (Adipogen) using the manufacturer’s protocol. Cultures were treated with an equal volume of media and an equal volume of supernatant was concentrated for each sample. Neuronal and astrocyte lysates (1 mg/mL) and concentrated supernatant were diluted in ELISA buffer as specified.

### qPCR

Total RNA was extracted from hiPSC, cortical neurons, and astrocytes using RNeasy Plus Mini Kit (Qiagen). cDNA was produced by reverse transcription using the High-Capacity RNA-to-cDNA Kit (Applied Biosystems, Thermo Fisher Scientific). To measure the endogenous gene expression of pluripotency factors and neuronal/astrocyte *GRN*, the qPCR analysis was performed using the 7900HT Fast Real-Time PCR System (Applied Biosystems, Thermo Fisher Scientific). mRNA was detected with Taqman probes (Thermo Fisher Scientific, *OCT4* Hs01895061_u1, *LIN28* Hs00702808_s1, *NANOG* Hs02387400_g1, *SOX2* Hs00602736_s1, *GRN* Hs00963707_g1, *HPRT1* Hs02800695_m1, and *GAPDH* Hs03929097_g1) in combination with the Taqman Universal PCR Master Mix (Applied Biosystems, Thermo Fisher Scientific). Gene expression of hiPSC markers was normalized to *GAPDH* housekeeping gene and compared to human fibroblast expression using the ΔΔCt-method. *GRN* gene expression in cortical neurons and astrocytes was normalized to the mean of *GAPDH* and *HPRT1* housekeeping genes and compared to vehicle-treated WT expression using the ΔΔCt-method.

### Trilineage differentiation

hiPSCs were differentiated into the three germ layers using the StemDiff™ Trilineage Differentiation Kit (STEMCELL Technologies) according to manufacturer’s instructions. After differentiation, cells were fixed in 4% PFA for 15 min for immunofluorescence analysis with anti-SOX1 (ectoderm), anti-SOX17 (endoderm), and anti-DESMIN (mesoderm) antibodies.

### AAV construct production and injections

The packaging of AAV-*GRN-*R493X-V5 was performed by the Petrucelli group at Mayo Clinic Jacksonville. The coding sequence for *GRN* V5-tagged R493X was cloned into the AAV expression vector pAM/CBA-EGFP-WPRE-BGH, AAV particles were packaged into serotype 9 capsid, and purified using standard methods [22]. Briefly, AAV was generated by co-transfection with the cis plasmids pF Delta6 and pRepCap9 into HEK293T cells. Cells were harvested 72 h after transfection, treated with 50 Units/ml Benzonase (Sigma-Aldrich), and lysed by freeze thaw. The virus (AAV-*GRN*-R493X-V5) was then purified using a discontinuous iodixanol gradient and buffer exchanged to PBS using an Amicon Ultra 100 Centrifugation device (EMD Millipore). The genomic titer of each virus was determined by quantitative PCR using the ABI 7700 (Applied Biosystems) and primers specific to the WPRE. The viral DNA samples were prepared for quantification by treating the virus with DNaseI (Invitrogen) and Proteinase K (Invitrogen), and samples were compared against a standard curve of supercoiled plasmid. AAV9-eGFP-Cre viral particles (AddGene, 105540-AAV9) were used to demonstrate technical proficiency through the detection of brain-wide human synapsin promoter driven eGFP expression in 10-week-old AAV9-eGFP-Cre mice. On the day of birth (P0), C57BL/6 neonates were anesthetized using isoflurane and bilaterally injected intracerebroventricularly with 0.5 μL of viral vector (AAV-*GRN*-R493X-V5 = 8E13 pfu/mL and AAV9-eGFP-Cre = 1E13 pfu/mL) through a finely drawn glass micropipette as described previously [23]. PTC readthrough treatment was performed when AAV-*GRN-*R493X-V5 mice were 6 weeks old.

### Surgical procedures

A single intracerebroventricular (ICV) bolus injection of vehicle, CDX5–288, or G418 ± CDX5–288 was performed in 6-week-old AAV-*GRN-*R493X-V5 mice according to previously published works with some modifications [24]. Mice lateral ventricles were injected stereotactically (3 μL volume, coordinates: − 1.0 mm lateral/− 0.3 mm posterior/− 3.0 mm depth to bregma) by loading a glass micropipette needle attached to a micro-syringe pump to deliver the dosage at a flow rate of 1 μL/min. Following drug injection, the needle was left in place for 3 min and then removed at a rate of 1 mm/second while holding a cotton swab against the skull at the base of the needle. The incision was then sutured with monocryl sutures (Ethicon, J303H) to close the skin. The mice were placed on the heating pad warmed half of a cage to ensure the mice were kept at 36–38 °C for the first 1.5 h while they recovered from the anesthesia, with the option for them to move away from the heat. After mice had recovered fully from surgery, they were transferred to the mouse housing room. 5 mg/kg meloxicam was injected subcutaneously once per day for the first 2 days following surgery.

### Brain collection and lysis

Seventy-two hours following ICV vehicle/drug injection, mice were perfused with PBS before brain collection. Brains were immediately flash frozen on dry ice. Whole brains were later thawed, homogenized in 500 μL TBS lysis buffer (150 mM NaCl, 1 mM EDTA, 1 mM sodium orthovanadate, 1 mM NaF, 1 mM β-glycerophosphate, 2.5 mM sodium pyrophosphate, 1 mM PMSF, PhosSTOP, cOmplete mini), sonicated for 10 s at 20% amplitude and ultracentrifuged at 100,000 x g for 20 min at 4 °C. The supernatants were collected as TBS-soluble extract, and the pellets were homogenized in RIPA buffer (TBS lysis buffer + 1% NP-40), sonicated, and again ultracentrifuged at 100,000 x g for 20 min at 4 °C. The supernatants were collected as RIPA-soluble extract. The protein concentration of RIPA-soluble fractions was measured by Bradford, diluted to 1 mg/mL and assayed by V5 WES analysis to detect AAV-*GRN-*R493X-V5 PTC readthrough products.

### Karyotyping analysis

Karyotyping was performed on WT, R418X^+/−^, & R493X^−/−^ KI by WiCell Cytogenetics, Inc. (Madison, WI). The S116X^+/−^ line was previously karyotyped by Cell Line Genetics (Madison, WI) following the production of this line [19].

### Statistical analysis

All values are expressed as the mean ± SEM. In experiments where two groups were compared a standard unpaired two-tailed Student’s *t*-test was performed to measure significance. For comparisons of more than two groups, one-way analysis of variance (ANOVA) was used followed by Tukey’s comparison post hoc test. *P-*values less than 0.05 were considered significant. Statistical analysis was performed using GraphPad Prism Software, Version 5.0.

## Results

### G418 combined with CDX5–288 maximizes *GRN* PTC readthrough in HEK293 cells

The efficiency of PTC readthrough is affected by the specific nonsense codon sequence and the flanking nucleotide sequences [25, 26]. Therefore, to survey the responsiveness of *GRN* nonsense mutations to PTC readthrough we designed several clinical *GRN* nonsense mutation expression constructs. We first transiently transfected HEK293 cells with S116X (UAA), R418X (UGA), and R493X (UGA) C-terminally V5-tagged expression constructs (Fig. 1a) and treated them for 72 h with the aminoglycoside G418. Mock transfected HEK293 cells showed no detectable V5 signal (Fig. S1). Since the V5 tag was inserted at the C-terminus, any V5 detected represented full-length PGRN generated by PTC readthrough. G418 induced *GRN* PTC readthrough in cells with the R418X and R493X mutations, with V5 (full-length PGRN) detected in both the cell lysate (intracellular) and supernatant (extracellular) fractions (Fig. S1). The accumulation of full-length PGRN in both the intra- and extracellular fractions of transiently transfected HEK293 cells suggests that the PGRN readthrough product retains its ability to be post-translationally processed via the secretory pathway. The S116X mutant did not respond to G418 treatment (Fig. S1), and this may be because aminoglycoside-induced PTC readthrough is most efficient at UGA nonsense codons and least efficient at UAA nonsense codons (UGA > UAG > UAA) [25].

To further screen and validate the *GRN* PTC readthrough observed in transiently transfected cells, we generated HEK293 lines stably expressing *GRN-V5* nonsense mutant constructs. When treated with G418 these cell lines exhibited a similar *GRN* PTC readthrough response to that observed in transiently transfected cells (Fig. 1b). Stable *GRN-V5* expressing HEK293 lines provided an ideal screening platform to test which compounds could most effectively enhance G418-induced *GRN* PTC readthrough. We treated HEK293 cell lines expressing nonsense mutant *GRN-V5* with G418 without or with readthrough enhancers CDX5–1 [14], CDX5–196 (Fig. S2a), or CDX5–288 (Fig. S2b) for 72 h, and measured intra- and extracellular V5 levels. CDX5 compounds enhanced G418-induced PTC readthrough activity in both the R418X and R493X mutant lines and full-length PGRN was detected in both the intra- and extracellular fractions (Fig. 1b). CDX5–288 produced the highest enhancement of readthrough activity (Fig. 1b). Thus, co-treatment with G418 and CDX5–288 was selected for further validation in patient hiPSC-derived cortical neurons and astrocytes and an AAV mouse model.

### FTLD-*GRN* patient hiPSC-derived cortical neurons and astrocytes show increased PGRN levels in response to PTC readthrough treatment

Next, we sought to determine whether treatment with G418 alone or in combination with CDX5–288 could rescue PGRN deficiency in more clinically relevant cell types with *GRN* heterozygosity or nullizygosity caused by nonsense mutations. An hiPSC line produced from an FTD-*GRN* patient bearing the g.585 C > A (S116X) *GRN* nonsense mutation (designated S116X^+/−^) was obtained from our stock [19]. Additionally, we generated hiPSC lines from one healthy control (designated WT) and an FTD-*GRN* patient carrying the g.2923 C > T (R418X) *GRN* nonsense mutation (designated R418X^+/−^) [18, 27]. We then used CRISPR/Cas9 gene-editing to generate from WT an isogenic clone homozygous for the most common FTD-*GRN* mutation g.3240 C > T (R493X) (designated R493X^−/−^ KI) (Fig. S3a-c). To characterize the hiPSC lines we assessed their expression of pluripotent markers at the protein and mRNA level, their ability to differentiate into all three germ layers, and demonstrated that they were all karyotypically normal (Fig. S4a,b).

**Fig. 2:**
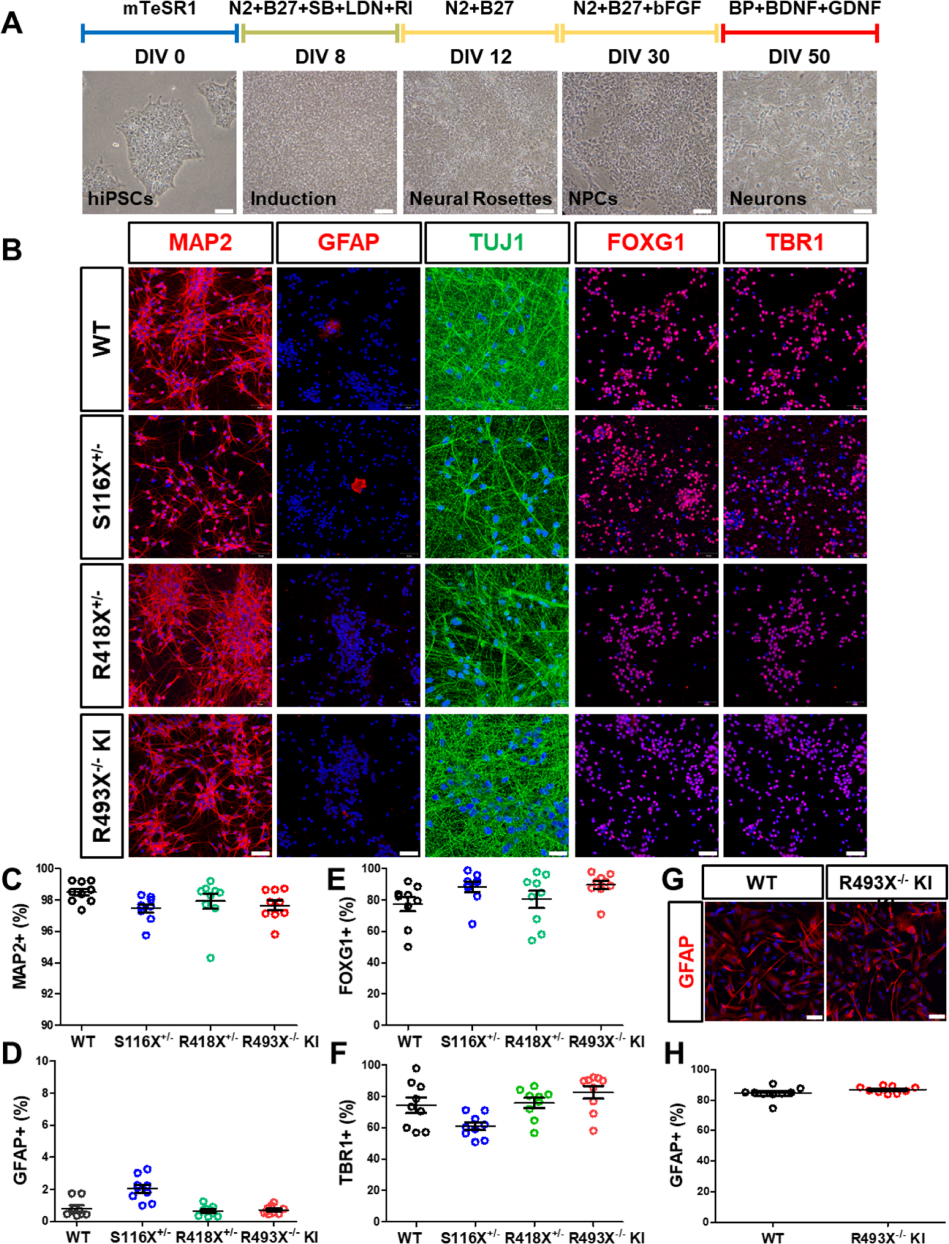
Differentiation and characterization of human cortical neurons and astrocytes derived from FTD-*GRN* patient and control hiPSCs. **a** Brightfield images of the cells at different stages of cortical neuronal differentiation. SB = SB 431542**,** LDN = LDN 193189, RI = Y-27632, BP = BrainPhys™, N2 = N-2 supplement, B27 = B-27 supplement. **b** Representative immunofluorescence images of DIV 50 WT, S116X^+/−^, R418X^+/−^, and R493X^−/−^ KI hiPSC-derived cortical neurons stained for MAP2, GFAP, TUJ1, FOXG1 (forebrain), and TBR1 (cortical layer VI). Cell nuclei were counterstained with DAPI (blue). Scale bar, 50 μm. **c**-**f** Cells positive for MAP2, GFAP, FOXG1, and TBR1 (neuronal, astrocytes, forebrain, and cortical layer VI markers, respectively) as a percentage of DAPI^+^ cells. On average, ~ 900 cells were analyzed per replicate, in (**c**-**f**) *n* = 3 independent cultures, 3 images per biological replicate; values are shown as mean ± SEM. **g** Representative immunofluorescence images of DIV 60+ WT and R493X^−/−^ KI hiPSC-derived astrocytes stained for GFAP. Cell nuclei were counterstained with DAPI (blue). Scale bar, 50 μm. **h** Cells positive for GFAP as a percentage of DAPI^+^ cells. On average, ~ 400 cells were analyzed per replicate, *n* = 3 independent cultures, 3 images per biological replicate; values are shown as mean ± SEM

We then differentiated the hiPSC lines into cortical neurons using a recently reported version of the dual SMAD inhibition neuroectoderm induction protocol [20] in combination with BrainPhys™ medium NPC neuronal maturation [21] (Fig. 2a). The resulting neuronal cultures possessed high levels of purity, as indicated by high percentage of MAP2 positive (> 95%) and low percentage of GFAP positive cells (< 4%) (Fig. 2b-d). Additionally, greater than 75% of cells expressed both FOXG1 and TBR1 confirming these neurons express cortical layer VI markers (Fig. 2b, e, f). Synaptogenesis was demonstrated in these cultures by staining for analysis of the expression of synapsin and excitatory/inhibitory synaptic markers (Fig. S5a, b). Astrocytes were also differentiated from NPCs using an astrocyte differentiation/maturation kit protocol. Mature astrocyte cultures (DIV 60+) derived from both WT and R493X^−/−^ KI hiPSC lines both expressed high levels (80%) of GFAP (Fig. 2g, h). Co-culturing neurons with WT human astrocytes on MEA plates led to the maturation of electrophysiologically active neurons and the formation of neural networks with frequent and robust spontaneous action potentials measured by MEA electrophysiology (Fig. S5c).

**Fig. 3:**
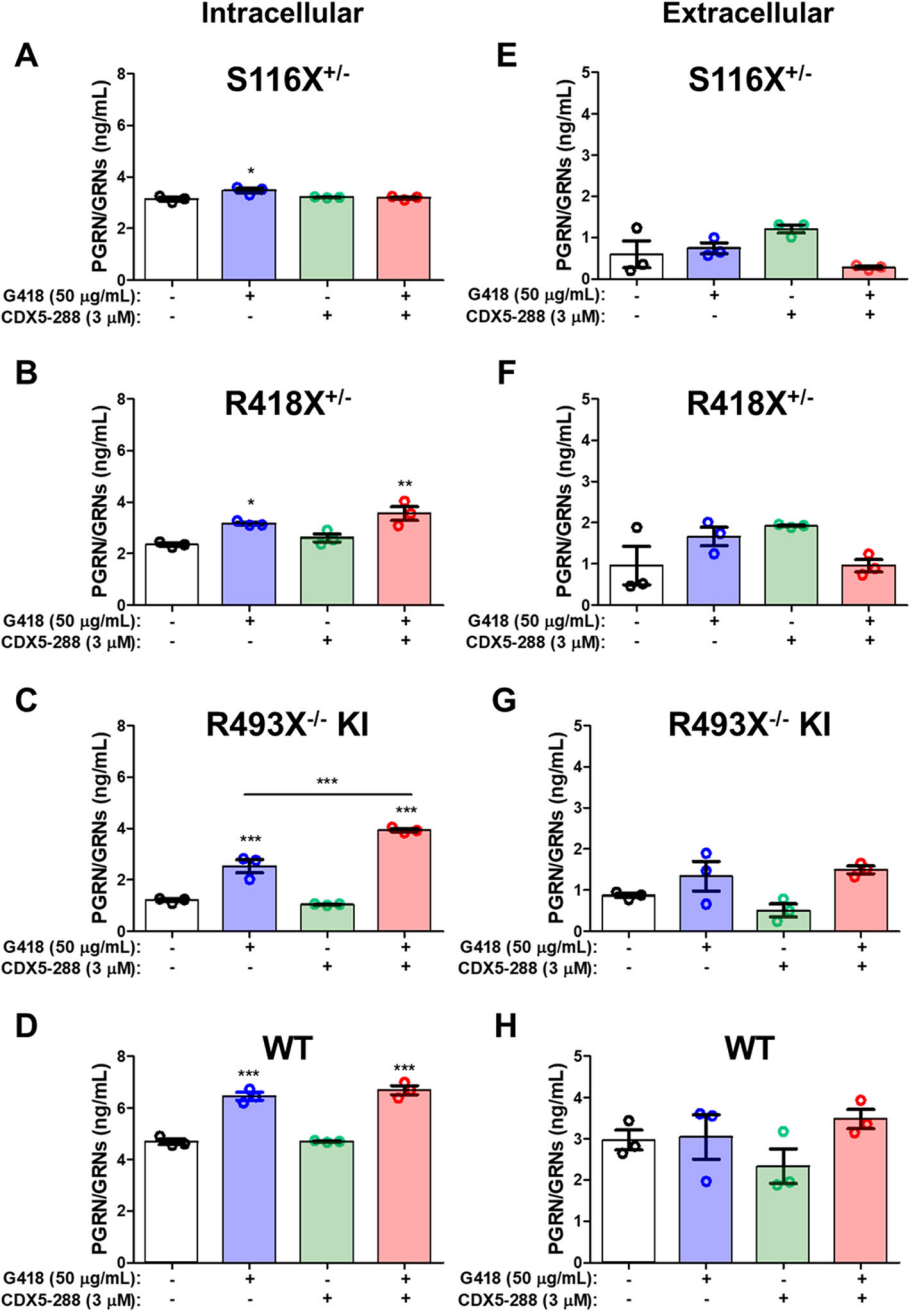
Induction of PTC readthrough by G418 and enhancers in hiPSC-derived cortical neurons bearing FTD-*GRN* nonsense mutations. DIV 50 S116X^+/−^, R418X^+/−^, R493X^−/−^ KI, and WT hiPSC-derived cortical neurons were treated with G418 and CDX5–288 at the indicated concentrations for 72 h. Intracellular (**a**-**d**, 1 mg/mL lysate) and extracellular (**e**-**h**, 25X concentrated supernatant) samples were subjected to PGRN ELISA using a polyclonal anti-PGRN antibody that targets multiple epitopes (GRN 1/3/5/7 peptides) and cannot differentiate between truncated/full-length PGRN or GRN peptides (PGRN/GRNs). *n* = 3 independent cultures; values are shown as mean ± SEM; * *p* < 0.05, ** *p* < 0.01, *** *p* < 0.0001 was determined by one-way ANOVA with Tukey’s multiple comparison test

hiPSC-derived cortical neurons were matured to DIV 50 and exposed to PTC readthrough compounds for 72 h. The levels of WT neuronal full-length PGRN expression were not sufficiently high for detection by western blotting with available anti-PGRN antibodies (Fig. S6, S7). As an alternative, intracellular and concentrated extracellular fractions were assayed by ELISA using a PGRN polyclonal antibody to multiple epitopes (GRN 1/3/5/7 peptides) that detects not only full-length PGRN but also truncated PGRN as well as granulin (GRN) peptides, collectively referred to here as PGRN/GRNs. Vehicle-treated S116X^+/−^, R418X^+/−^, and R493X^−/−^ KI neurons expressed 33.1% ± 1.5, 49.9% ± 1.3, and 74.5% ± 1.2% less intracellular PGRN/GRNs than vehicle-treated WT neurons, as expected (Fig. 3, S8). In general, treatment of FTLD-*GRN* mutant neurons with G418 without or with CDX5–288 produced a similar pattern of PGRN/GRNs expression to that observed in the stably transfected HEK293 cell lines bearing the same mutations. Again, the S116X^+/−^ neurons exhibited little to no intra- or extracellular increase in the levels of PGRN/GRNs in response to PTC readthrough treatment (Fig. 3a, e). R418X^+/−^ neurons exposed to G418 alone or to G418 and CDX5–288 showed significantly increased intracellular PGRN/GRNs levels to 67.5% ± 1.2 and 75.8% ± 5.8% of vehicle-treated WT levels, respectively (Fig. 3b). Treatment of R493X^−/−^ KI neurons with G418 alone significantly increased intracellular PGRN/GRNs levels, to 54.0% ± 5.5% of vehicle-treated WT levels and exposure to G418 and CDX5–288 further significantly increased intracellular PGRN/GRNs levels compared G418 alone, to 83.9% ± 1.3% of vehicle-treated WT (Fig. 3c). For both R418X^+/−^ neurons and R493X^−/−^ KI neurons, extracellular PGRN/GRNs levels were not significantly increased in response to treatment although a trend toward a minor increase was observed (Fig. 3f, g). Since R493X^−/−^ KI neurons have no WT *GRN* allele, the observed increase in intracellular PGRN/GRNs is likely due to expression of the mutated allele. G418 did not show significant cellular toxicity in iPSC-derived neurons (Fig. S9).

Contrary to expectation, we observed that WT cortical neurons treated with G418 alone and in combination with CDX5–288 showed significantly increased intracellular PGRN/GRNs levels relative to vehicle-treated cells, by 37.9% ± 3.2 and 42.9% ± 3.7, respectively (Fig. 3d). This intracellular increase in WT neuronal PGRN/GRNs levels was not due to PTC readthrough as both *GRN* alleles are WT. CDX5–288 alone did not increase intracellular WT neuronal PGRN/GRNs and its combination with G418 did not increase PGRN/GRNs over G418 treatment alone, indicating the observed increase is mediated by G418 (Fig. 3d). The G418-mediated increase in intracellular WT neuronal PGRN/GRNs expression was not accompanied by a corresponding increase in PGRN/GRNs secretion (Fig. 3h). These findings in WT neurons suggest G418 may disrupt PGRN exocytosis. The potential mechanisms driving this phenomenon are addressed in more detail in the context of WT astrocytes (Fig. 5, 6b).

**Fig. 4:**
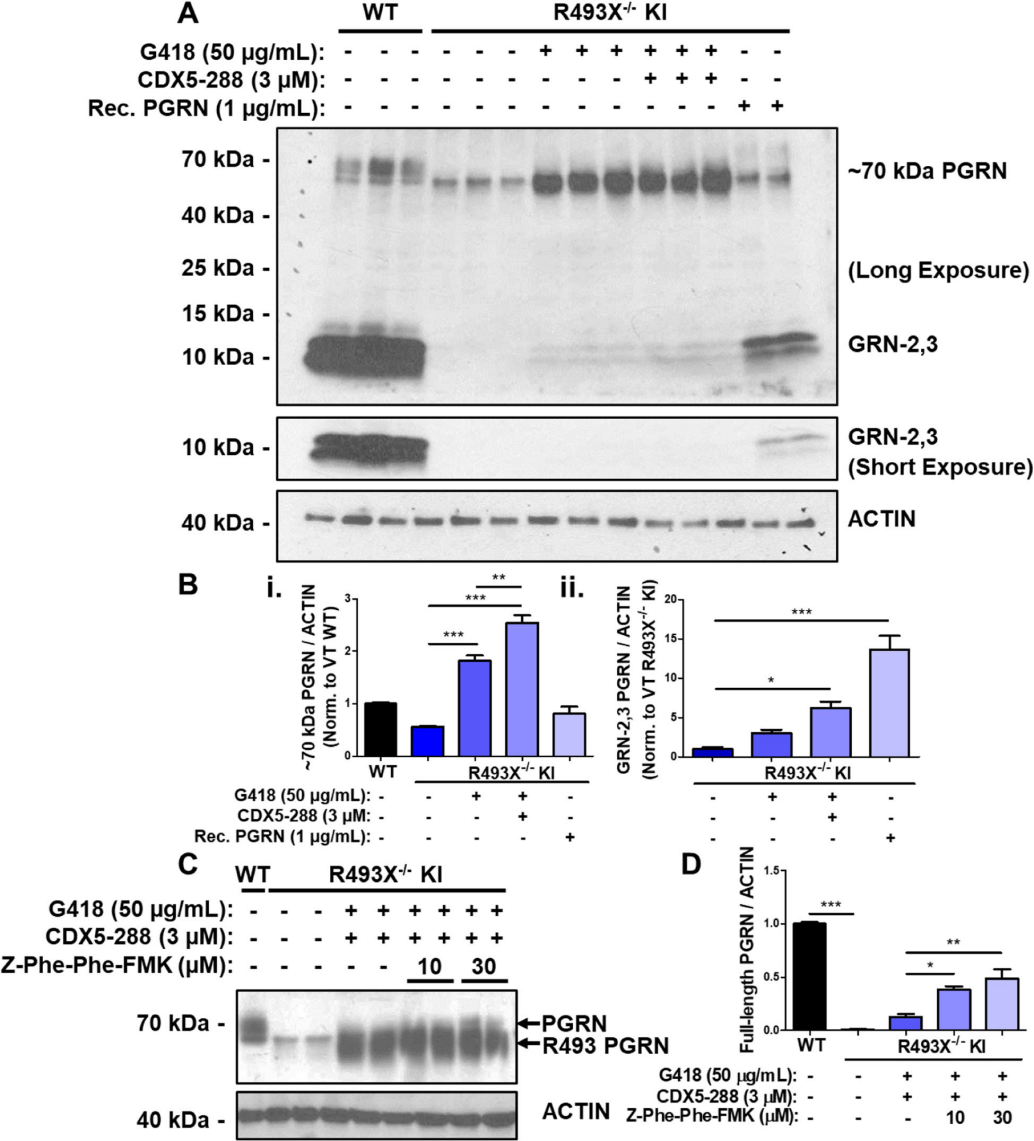
Induction of PTC readthrough by G418 and enhancers in hiPSC-derived R493X^−/−^ KI astrocytes. **a** R493X^−/−^ KI hiPSC-derived astrocytes were treated with vehicle solution, G418 alone, G418 in combination with CDX5–288, and rec. Human PGRN at the indicated concentrations for 72 h. Expression of PGRN and GRN-2,3 peptides in treated WT and R493X^−/−^ KI astrocyte samples were analyzed by western blotting, using actin as the loading control. **b** Densitometric quantification of ~ 70 kDa PGRN (***i***) and GRN-2,3 peptide (***ii***) in astrocyte lysates (**a**) normalized to vehicle-treated (VT) WT levels. VT WT was excluded from ***ii*** due to oversaturation of GRN-2,3 signal in long exposure blot. For clarity, rec. Human PGRN treated R493X^−/−^ KI astrocytes expressed 20.9% ± 0.027 of VT WT GRN-2,3 levels based on quantification of the short exposure blot (data not shown). **c** R493X^−/−^ KI hiPSC-derived astrocytes were treated with vehicle solution, G418 in combination with CDX5–288, and G418 CDX5–288 combination with either 10 or 30 μM of Z-Phe-Phe-FMK for 72 h. Again, expression of PGRN in WT and R493X^−/−^ KI astrocyte lysates was also analyzed by western blotting, using actin as the loading control. **d** Densitometric quantification of full-length PGRN in astrocyte lysates (**c**) normalized to VT WT levels. *n* = 3 independent cultures (except in **d***n* = 2); values are shown as mean ± SEM; *p* < 0.05, ** *p* < 0.01, *** *p* < 0.0001 was determined by one-way ANOVA with Tukey’s multiple comparison test

Next, WT and R493X^−/−^ KI hiPSC-derived astrocytes were matured to DIV 60+ to extend testing of readthrough compounds to another relevant CNS cell type with endogenous nonsense mutant *GRN* expression. WT hiPSC-derived astrocytes produced 17.9-fold more intracellular PGRN/GRNs, secreted 25.0-fold more PGRN/GRNs, and expressed 3.7-fold higher *GRN* mRNA than WT hiPSC-derived neurons as measured by ELISA and qPCR (Fig. S6). This considerably higher expression level enabled analysis of intracellular PGRN levels by western blotting in hiPSC-derived astrocyte cultures (Fig. 4). The PGRN antibody used in this western blot analysis has previously been shown to detect full-length PGRN as well as GRN peptides (GRN-2,3) [28]. In WT astrocytes, both PGRN and GRN-2,3 peptides were detected, though the GRN-2,3 peptides were more abundant than full-length PGRN (Fig. 4a). R493X^−/−^ KI astrocytes expressed small amounts of a band at ~ 70 kDa that may be PGRN truncated at R493, and no detectable GRN-2,3 peptides (Fig. 4a). Treating R493X^−/−^ KI astrocytes with either G418 alone or G418 and CDX5–288 significantly increased intracellular ~ 70 kDa PGRN, by 3.3- and 4.6-fold, respectively (Fig. 4a, b.i). This increase in intracellular ~ 70 kDa PGRN was accompanied by a small increase in GRN-2,3 peptide signal, which indicates that at least some of the protein entered lysosomes and was cleaved into individual GRN peptides (Fig. 4a, b.ii). Exposing R493X^−/−^ KI astrocytes to extracellular recombinant human PGRN showed that these cells can take up and process PGRN, as the vast majority of endocytosed full-length PGRN was converted into GRN peptides (Fig. 4a, b). Since the rate of conversion of full-length PGRN to GRN-2,3 peptides is so efficient in astrocytes, we hypothesized that full-length PGRN generated by readthrough would be rapidly processed into GRN peptides, perhaps explaining why we observe limited full-length PGRN in G418 and CDX5–288 combination-treated R493X^−/−^ KI astrocytes. The lysosomal cysteine protease cathepsin L cleaves PGRN into GRN peptides [29]. Therefore, we tested whether inhibiting cathepsin L during G418 and CDX5–288 induced PTC readthrough in R493X^−/−^ KI astrocytes could enable clear visualization of full-length PGRN by western blot. Co-treating R493X^−/−^ KI astrocytes with 30 μM Z-Phe-Phe-FMK (Cathepsin L inhibitor), G418 and CDX5–288 led to significant accumulation of PTC readthrough derived full-length PGRN (Fig. 4c, d), confirming our hypothesis.

**Fig. 5:**
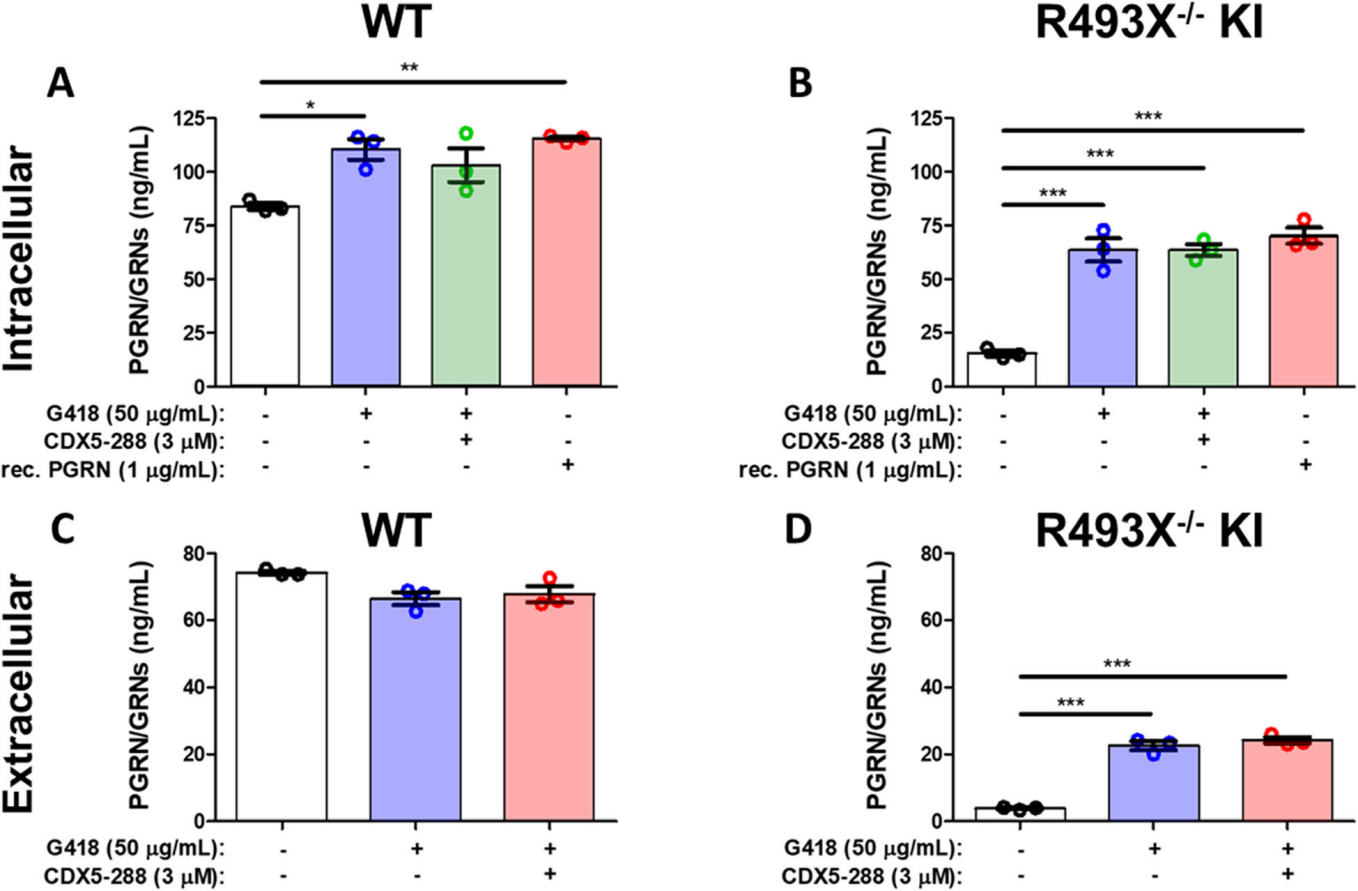
Induction of PTC readthrough by G418 and enhancers in R493X^−/−^ KI hiPSC-derived astrocytes. DIV 60+ WT and R493X^−/−^ KI hiPSC-derived astrocytes were treated with G418, CDX5–288, and rec. Human PGRN at the indicated concentrations for 72 h. Intracellular (**a**-**b**, 1 mg/mL lysate) and extracellular (**c**-**d**, 25X concentrated supernatant) samples were subjected to PGRN ELISA using a polyclonal anti-PGRN antibody that targets multiple epitopes (GRN 1/3/5/7 peptides). Therefore, the antibody cannot differentiate between truncated/full-length PGRN or GRN peptides (PGRN/GRNs). *n* = 3 independent cultures; values are shown as mean ± SEM; * *p* < 0.05, ** *p* < 0.01, *** *p* < 0.0001 was determined by one-way ANOVA with Tukey’s multiple comparison test

**Fig. 6:**
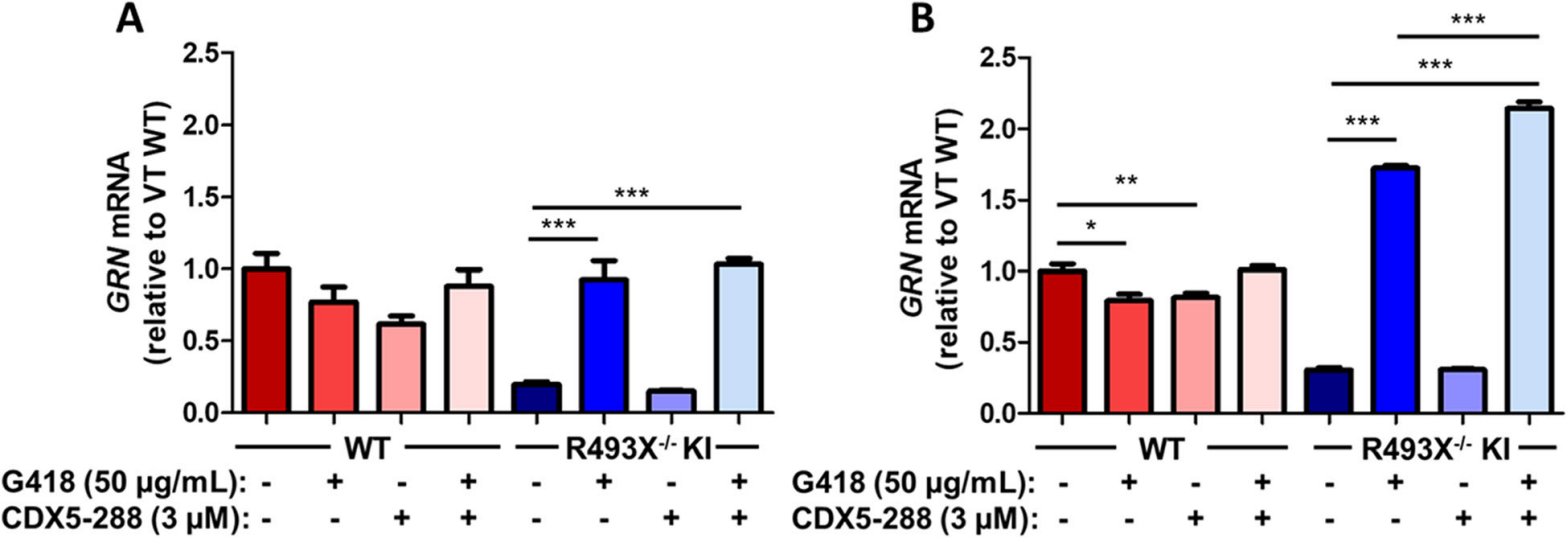
Selective increase of R493X^−/−^ KI nonsense mutant mRNA by PTC readthrough treatment. qPCR analysis of human *GRN* mRNA levels in DIV 50 WT and R493X^−/−^ KI hiPSC-derived cortical neurons (**a**) and DIV60+ astrocytes (**b**) treated with G418 and CDX5–288 at the indicated concentrations for 72 h. Relative *GRN* mRNA levels were normalized to the mean of *GAPDH* and *HPRT1* housekeeping genes and compared to vehicle-treated (VT) WT. *n* = 3 independent cultures; values are shown as mean ± SEM; * *p* < 0.05, ** *p* < 0.01, *** *p* < 0.0001 was determined by one-way ANOVA with Tukey’s multiple comparison test

We next used ELISA to measure of intra- and extracellular PGRN/GRNs levels in astrocytes. Vehicle-treated R493X^−/−^ KI astrocytes expressed 81.8% ± 1.6% less intracellular PGRN/GRNs than vehicle-treated WT astrocytes (Fig. 5a, b). R493X^−/−^ KI astrocytes exposed to G418 alone or G418 and CDX5–288 showed significantly increased intracellular PGRN/GRNs levels, to 75.7% ± 6.5 and 75.8% ± 3.2%, respectively, of vehicle-treated WT levels, only slightly less than PGRN/GRNs restoration achieved through the application of exogenous recombinant human PGRN (Fig. 5b). Vehicle-treated R493X^−/−^ KI astrocytes secreted 94.9% ± 0.3% less PGRN/GRNs than vehicle-treated WT astrocytes (Fig. 5c, d). Exposure to G418 alone or G418 and CDX5–288 caused a considerable increase in secreted PGRN/GRNs, to 30.4% ± 1.7 and 32.5% ± 1.2%, respectively, of vehicle-treated WT levels (Fig. 5c, d), unlike R493X^−/−^ KI cortical neurons, where treatment increased intracellular PGRN/GRNs but only subtly increased PGRN/GRNs secretion. G418 increased intracellular PGRN/GRNs in WT astrocytes by 31.7% ± 5.6%, without a corresponding increase in secreted PGRN/GRNs (Fig. 5a, c), as was observed with WT cortical neurons. Western blotting revealed that G418 significantly increased the intracellular levels of PGRN while decreasing the levels of GRN-2,3 peptides in WT astrocytes (Fig. S10). Therefore, G418 may disrupt the normally highly efficient lysosomal processing of full-length PGRN to GRN peptides in astrocytes. To further probe the mechanism of this unanticipated readthrough-independent increase in WT intracellular PGRN, we conducted *GRN* qPCR analysis in WT hiPSC-derived neurons and astrocytes. Treating WT neurons or astrocytes with G418 alone or in combination with CDX5–288 did not significantly increase *GRN* mRNA levels (Fig. 6a, b). We speculate that G418 affects PGRN homeostasis at the protein level resulting in reduced lysosomal processing of full-length PGRN into individual GRN peptides.

qPCR analysis revealed that readthrough compounds caused a strong increase in *GRN* mRNA levels in R493X^−/−^ KI neurons and astrocytes, in contrast to WT cells. This was an anticipated observation as mRNAs bearing nonsense mutations are targeted for degradation by nonsense-mediated mRNA decay (NMD) [30] and PTC readthrough enables escape of nonsense mutant mRNA from degradation by NMD. Vehicle-treated R493X^−/−^ KI neurons expressed 5.1-fold less *GRN* mRNA relative to vehicle-treated WT neurons (Fig. 6a), and vehicle-treated R493X^−/−^ KI astrocytes expressed 3.3-fold less *GRN* mRNA than vehicle-treated WT astrocytes (Fig. 6b), indicating downregulation of mutant mRNA by NMD. When treated with G418 or combination, R493X^−/−^ KI neurons and astrocytes showed large increases in *GRN* mRNA (Fig. 6a, b). The selective stabilization of PTC-bearing *GRN* mRNA over WT *GRN* mRNA strongly supports inhibition of NMD consequent to readthrough in R493X^−/−^ KI neurons and astrocytes. Taken together these results show that the G418 - CDX5–288 combination induces PTC readthrough and effectively restores intracellular levels of PGRN in both heterozygous and homozygous *GRN* nonsense mutant neurons and astrocytes to levels approaching that of healthy control cells, while also revealing that G418 can interfere with intracellular PGRN processing.

### Restoration of PGRN levels by PTC readthrough rescues lysosomal dysfunction in FTD-R493X^−/−^ KI hiPSC-derived cortical neurons

The specific nonsense mutant codon (ex: R493X^−/−^ KI, Arg-CGA to X-UGA) restricts which near-cognate aminoacyl-tRNA molecules (Trp UGG, Cys UGC, Arg CGA, etc.) pair with the PTC in the ribosome A site and contribute their amino acid to the growing polypeptide during a readthrough event. Previous studies have demonstrated that the amino acids most commonly incorporated at UGA nonsense mutations during readthrough were Trp, followed by Cys and Arg [31]. Thus, it is likely a proportion of full-length PGRN derived from *GRN* R493X^−/−^ KI PTC readthrough would possess a missense mutation at codon 493. Therefore, it was important to assess the functionality of PGRN produced by PTC readthrough, through its ability to rescue known cellular FTLD-*GRN* and NCL phenotypes.

**Fig. 7:**
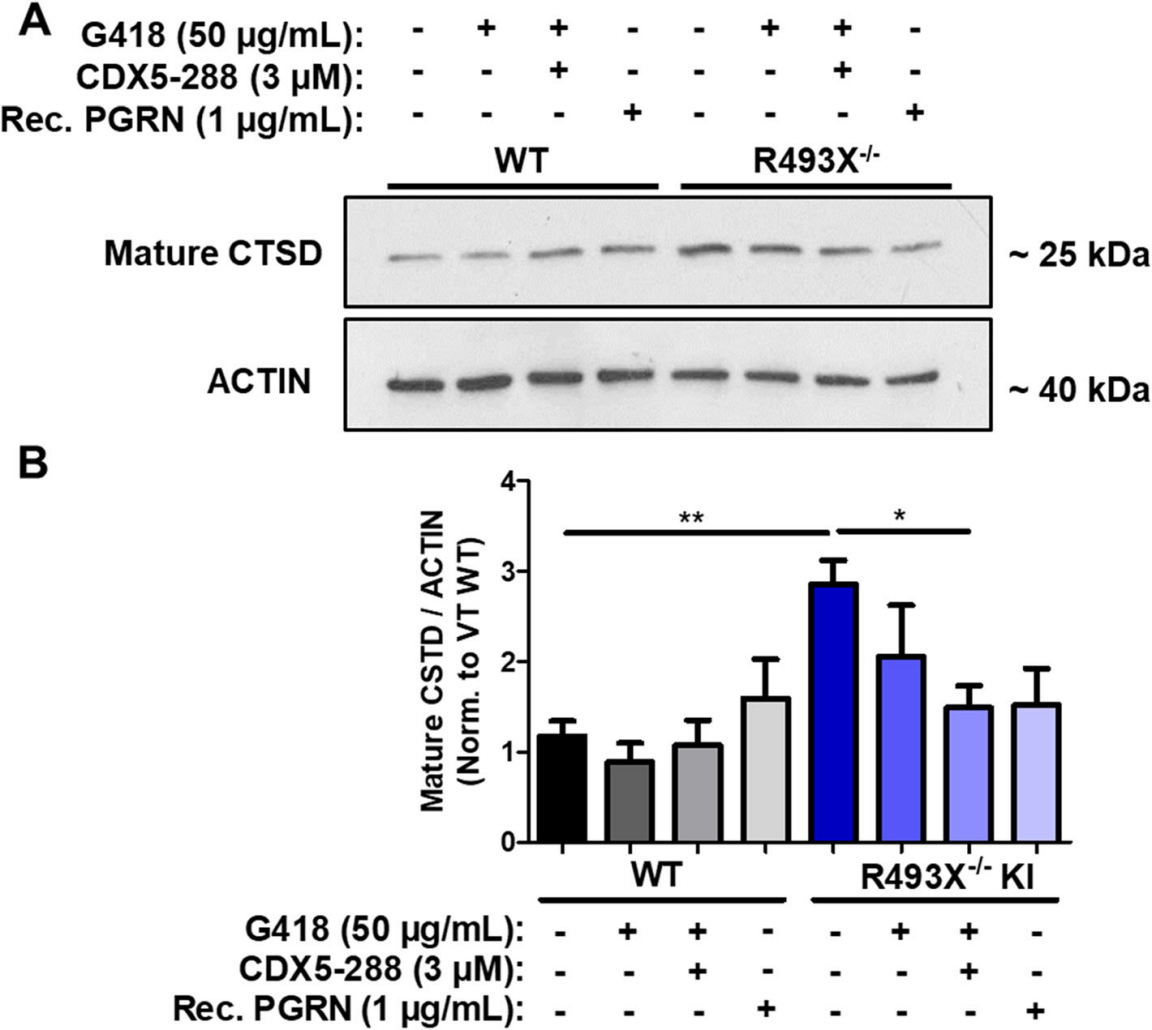
*GRN* PTC readthrough rescues FTLD/NCL lysosomal pathological CSTD maturation phenotype in hiPSC-derived R493X^−/−^ KI cortical neurons. **a** DIV 80 WT and R493X^−/−^ KI hiPSC-derived cortical neurons were treated with vehicle solution, G418 alone, G418 in combination with CDX5–288, and rec. Human PGRN at the indicated concentrations for 72 h. Expression of mature CSTD in treated WT and R493X^−/−^ KI cortical neuron lysates analyzed by western blotting, using actin as the loading control. **b** Densitometric quantification of CSTD expression in the aforementioned cortical neuron lysates normalized to vehicle-treated WT levels. *n* = 3–6 independent cultures; values are shown as mean ± SEM; * *p* < 0.05, ** *p* < 0.01, was determined by one-way ANOVA with Tukey’s multiple comparison test

Lysosomal maturation defects have recently been demonstrated as one of the earliest disease phenotypes that arise in mice null for *Grn* [15, 17]. *Grn*^−/−^ mouse brains upregulate the expression, maturation rate, and catalytic activity of cathepsin lysosomal proteases (cathepsin D, B, and L) in an age-dependent manner [17], and this aberrant lysosomal phenotype has been previously rescued in vivo by treating aged *Grn*^−/−^ mice with AAV-mediated *Grn* gene therapy [7]. Moreover, hiPSC-derived neurons with heterozygous *GRN* mutations have been shown to exhibit an increased CTSD maturation phenotype [16]. We quantified the CTSD levels in aged (DIV 80) WT and R493X^−/−^ KI hiPSC-derived cortical neurons and observed significantly increased expression of the mature form of CTSD in vehicle-treated R493X^−/−^ KI neurons compared with WT neurons (Fig. 7a, b). Treating WT neurons with G418, G418 + CDX5–288, or recombinant human PGRN did not affect mature CTSD expression levels (Fig. 7a, b). However, treating R493X^−/−^ KI neurons with G418 and CDX5–288 significantly reduced mature CTSD levels, thus rescuing their dysregulated lysosomal enzyme phenotype. Importantly, this effect was also observed in R493X^−/−^ KI neurons treated with extracellular recombinant human PGRN with a trend towards reduced mature CTSD levels (Fig. 7a, b). These findings provide evidence that PGRN expression restored by PTC-readthrough in R493X^−/−^ KI cortical neurons (Fig. 3d) is biologically active and functional in vitro.

### Intracerebroventricular administration of G418 induces *GRN* PTC readthrough in adult AAV mice expressing a *GRN*-R493X-V5 construct

**Fig. 8:**
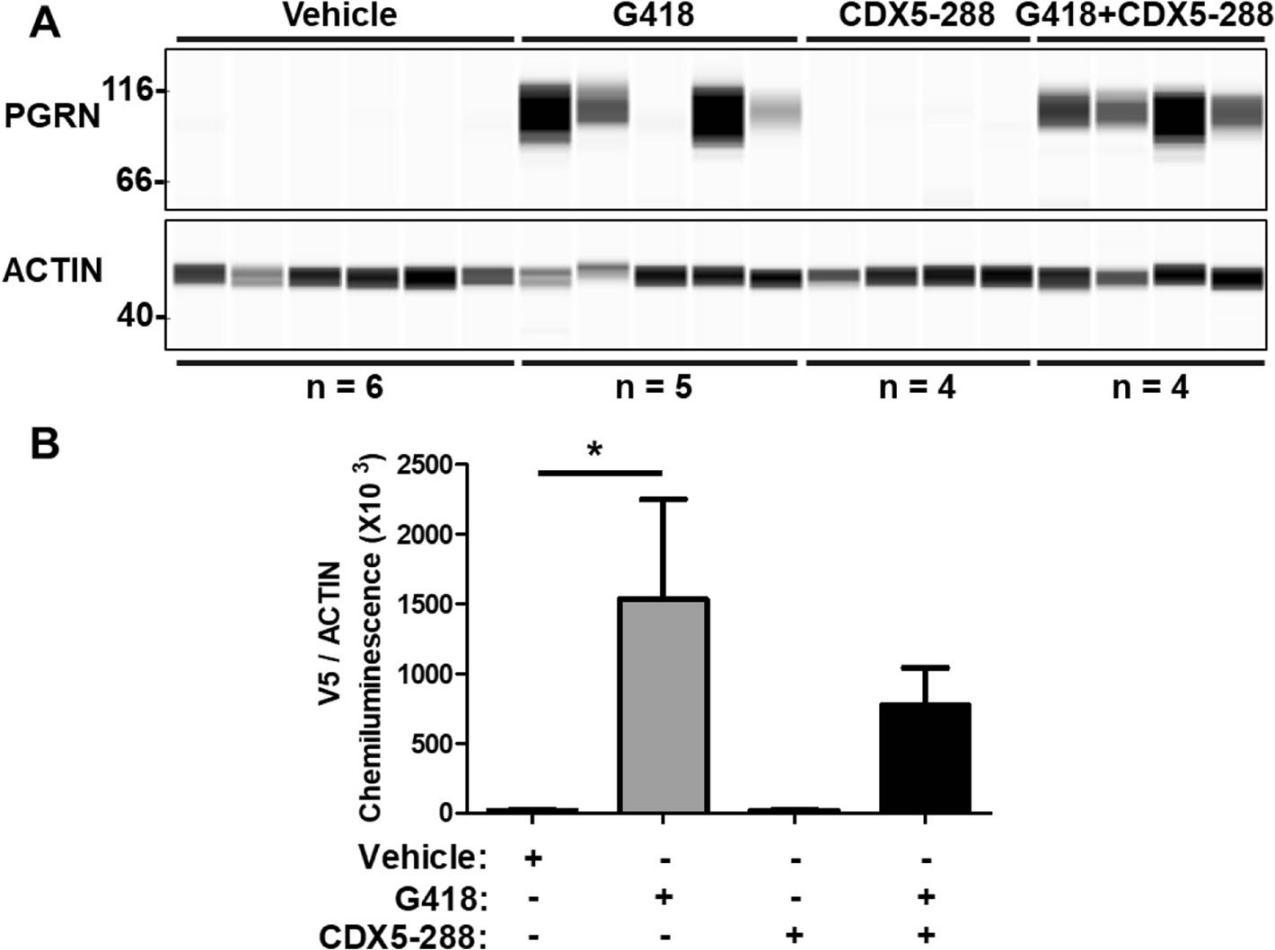
Induction of PTC readthrough in the mouse brain expressing R493X *GRN-V5*. **a** 6-week-old AAV-*GRN*-R493X-V5 mice were stereotactically injected ICV with either vehicle solution, G418, CDX5–288, or a combination of G418 + CDX5–288 and sacrificed after 72 h. Whole-brain RIPA-soluble protein extracts were subjected to automated capillary electrophoresis western analysis. Full-length PGRN was detected with a V5 antibody and actin was measured in brain lysates as a loading control. **b** Chemiluminescence quantification of automated capillary electrophoresis western analysis V5 detection normalized to actin. Values are shown as mean ± SEM; * *p* < 0.05 was determined by one-way ANOVA with Tukey’s multiple comparison test

Having demonstrated PTC readthrough in disease-relevant cellular models of FTLD-*GRN*, we sought to provide initial proof-of-concept for this approach in vivo. CNS ICV PTC readthrough induced by G418 has been previously demonstrated in mice harboring an inducible gene-targeting system driven by the full-length expression of a nonsense mutant Cre recombinase [32]. We generated AAV particles containing the same *GRN*-*V5* R493X reading frame used for HEK293 transfections. Since this plasmid does not contain a reporter gene, to establish that our AAV injection technique could achieve an adequate percentage of neuronal transduction we first tested an AAV9-eGFP-Cre vector and observed diffuse brain-wide eGFP expression (Fig. S11). Next, newborn pups (P0) were bilaterally injected ICV with virus solution and aged to at least 6 weeks to allow for sufficient blood-brain-barrier maturation before treatment [33]. RIPA-soluble brain lysates from each ICV treated AAV-*GRN*-R493X-V5 mouse were probed by for V5 expression to assess the extent of *GRN* R493X PTC readthrough in vivo. No V5 signal was detected in either the vehicle (*n* = 6) or CDX5–288 (*n* = 4) AAV-*GRN*-R493X-V5 brains 72 h after ICV injection (Fig. 8a, b). By contrast, ICV injections with G418 alone (*n* = 5) or G418 and CDX5–288 (*n* = 4) induced clear *GRN*-R493X-V5 PTC readthrough, as shown by the detection of V5 signal at the molecular weight (MW) of PGRN, using automated western analysis (WES) (Fig. 8a, b). G418 significantly induced readthrough in AAV-*GRN*-R493X-V5 mice compared to treatment with vehicle solution (Fig. 8a, b). Though robust V5 levels were detected, co-treatment of AAV-*GRN*-R493X-V5 mice with CDX5–288 did not increase full-length PGRN levels compared to G418 alone (Fig. 8a, b). Our data indicate that the aminoglycoside G418 can induce *GRN* PTC readthrough in vivo when delivered intraventricularly in an exogenous AAV transgene expression model.

## Discussion

In this study, we show that PGRN insufficiency caused by *GRN* nonsense mutations can be ameliorated through PTC readthrough in in vitro and in vivo models of FTLD-*GRN*, and that the resultant increase in PGRN reverses FTLD-*GRN*-related lysosomal dysfunction. We first generated several human expression constructs with known clinical *GRN* nonsense mutations, and observed the most robust PTC readthrough in response to G418 and CDX5–288 enhancer in R418X and R493X mutant HEK293 cells compared to G418 treatment alone or co-treatment with other enhancer compounds. To avoid confounding measures of endogenous PGRN in HEK293 cells, and to ensure the measured PGRN was reflective of full-length protein produced by readthrough, the *GRN* expression constructs bore a C-terminal V5 tag. The readthrough enhancer compounds are N-substituted phthalimide derivatives that were identified in a high throughput screen in combination with sub-active concentrations of the aminoglycoside paromomycin, and further systematically functionalized to increase their potency [14]. To verify *GRN* PTC-readthrough in a more disease-specific context, we tested the same G418/enhancer combination in cortical neurons differentiated from patient-derived iPSCs bearing the *GRN* nonsense mutations tested in HEK293 cells. Similar to our results in HEK293 cells, *GRN* readthrough and up to 2.5X increase in PGRN/GRNs was observed in neurons with the R418X (heterozygous) and R493X (homozygous) nonsense mutations after 72 h exposure to G418 and CDX5–288 combination treatment. In both HEK293 cells as well as human neurons we did not observe readthrough in cells bearing the S116X nonsense mutation, most consistent with less efficient readthrough previously reported with TAA stop codons [25].

Since the *GRN* expression constructs in HEK293 cells bore a C-terminal V5 tag, the resulting PGRN could only be explained by *GRN* readthrough yielding full-length protein. In human neurons we were not able to detect full-length PGRN, likely due to their lower levels of expression or rapid processing into smaller granulin peptides (Fig S6, S7). Therefore, we used a commercially available ELISA kit that detects PGRN at multiple epitopes (GRN-1, 3, 5, & 7) to measure general changes in cortical neuron PGRN expression, precluding discrimination between the truncated and full-length forms of PGRN or GRN peptides. In contrast, we showed that full-length and truncated PGRN are measurable by western blot in human astrocytes, allowing more precise measurements of PGRN protein of various sizes, including full-length, truncated forms, and GRN peptides. In response to G418 and CDX5–288, there is a significant increase in truncated and likely full-length PGRN in R493X^−/−^ KI astrocytes, as well as an increase in GRN peptides 2,3. This increase reflects a greater than 6-fold increase in *GRN* mRNA in response to G418 and CDX5–288 combination treatment. Increased mRNA is characteristic of escape from NMD, in which nonsense mutant mRNA undergoes its pioneering round of full-length translation without stalling at its PTC. This process is thought to be regulated by the degree of PTC readthrough, and only a minor increase in translation beyond a proposed 0.5% threshold would be sufficient for substantial NMD inhibition and increased translation as shown in yeast [34]. G418 has been previously demonstrated to inhibit NMD through this readthrough-dependent mechanism [35]. Stabilization of R493X mutant *GRN* mRNA leads to accumulation of truncated protein, which was evident in R493X^−/−^ KI astrocytes treated with both G418 alone and CDX5–288 combination. Moreover, the increase in ~ 70 kDa PGRN signal detected in the R493X^−/−^ KI astrocytes was accompanied by a faint slightly higher MW band (Fig. 4a) that we suspected may represent full-length PGRN. Given that both WT and R493X^−/−^ KI astrocytes rapidly and efficiently process PGRN into individual GRN peptides it is likely that the majority of PTC readthrough-derived full-length PGRN would also be cleaved into GRNs, thus making full-length PGRN particularly difficult to detect. This hypothesis is supported by the observed increase in full-length PGRN in R493X^−/−^ KI astrocytes in response to readthrough treatment, when the major lysosomal PGRN processing enzyme cathepsin L is inhibited. These results suggest that the increase in intracellular PGRN/GRNs in R493X^−/−^ KI cortical neurons likely reflects a mixture of both truncated and full-length PGRN.

Interestingly, treating R493X^−/−^ KI cortical neurons with G418 and CDX5–288 combination rescued aberrant lysosomal function in these cells, reducing mature CTSD levels back to WT levels. Recent studies have discovered a physical interaction between PGRNs C-terminus and CTSD [16, 36, 37]. Despite some conflicting reports [17], the growing consensus is that PGRN binds to the pro-form of CTSD promoting its maturation, thus increasing its enzymatic activity [16, 38]. However, this hypothesis is contradicted by the observation that mature CSTD is upregulated in *Grn*^*−/−*^ mice brains/microglia and can be rescued with the addition of exogenous mouse recombinant Pgrn [7, 39]. Nevertheless, our results suggest that PGRN generated by PTC RT can perform biological functions, and provides supplementary validation for at least a portion of the readthrough derived neuronal PGRN being full-length. It has also been recently demonstrated that the truncated PGRN R493X protein retains some of its biological properties, such as lysosomal localization and an ability to suppress a proinflammatory immune response in *Grn*^*−/−*^ mice bone marrow-derived macrophage cultures [40]. Therefore, we cannot rule out a contribution of truncated PGRN in the correction of this excess mature CTSD phenotype.

Unexpectedly, G418 induced accumulation of WT endogenous intracellular PGRN in all of our in vitro models. Given that G418 increases WT PGRN levels, it was difficult to prove that PTC readthrough was responsible for the elevated PGRN expression detected in heterozygous R418X^+/−^ cortical neurons, as any increase in PGRN could be attributable to either increased expression from the WT allele or PTC-readthrough of the nonsense mutant allele. This provided the rationale for developing a homozygous R493X^−/−^ KI line to address this issue and establish that at least a portion of the increased PGRN expression we observed in R418X^+/−^ neurons was likely reflective of PTC readthrough. *GRN* expression analysis in WT cortical neurons and astrocytes treated with G418 confirmed that the increase in intracellular PGRN is not due to an accumulation of *GRN* mRNA, suggesting the mechanism is post translational in nature. The G418-mediated intracellular PGRN increase in WT neurons and astrocytes does not result in corresponding accumulation of PGRN in culture media, implying that G418 may interfere with the PGRN secretory pathway. In R493X mutant astrocytes, treatment with G418 with or without enhancer compound resulted in a more pronounced increase in both mRNA and PGRN than that observed in neurons. We cannot fully rule out G418-related mechanisms other than readthrough that may uniquely impact PGRN processing in astrocytes. Although G418 is not suitable for human use, further studies would be needed to better understand a possible role for G418 in PGRN processing.

As G418 and other aminoglycosides do not readily cross the blood-brain barrier, we sought to determine whether ICV administration of compounds could be a viable strategy for widespread brain exposure and readthrough. Indeed, our results provide proof-of-concept that a single ICV injection of G418 can induce robust PTC readthrough in an AAV-*GRN*-R493X-V5 mouse model in only 72 h. Given the experimental complexity associated with drug screening in AAV models, which likely contributed to relatively high variability seen in injected mice, we were unable to identify an optimal dosage of enhancer compound to treat in combination with G418. The newly available R493X KI mouse model of FTLD-*GRN*/NCL [40] may be a better and more consistent model to further explore the optimal in vivo doses of both G418 and enhancer compounds.

To our knowledge this is the first report demonstrating the ability of nonsense mutation readthrough to enhance PGRN expression in FTLD-*GRN* patient-derived neurons/astrocytes, as well as in an in vivo AAV mouse model expressing a human *GRN* construct with the most common R493X pathogenic mutation. Since the submission of our work, another group have demonstrated *GRN* readthrough in response to G418 in a mouse neuroblastoma cell line (N2A) overexpressing a human *GRN* R493X construct [41]. This preliminary report further validates our current results in human cell lines. Importantly, there are several known clinical limitations with the use of aminoglycosides as a potential therapeutic [42–44]. While G418 is not suitable for long-term human consumption, our work shows that PTC readthrough is a promising strategy in FTLD-*GRN*, and highlights the importance of ongoing and future efforts to find novel readthrough compounds more suitable for human use. Our work also shows a narrow therapeutic window, such as that seen with gentamicin, can be mitigated through enhancer compounds. Future studies are needed to further explore whether PTC readthrough induced PGRN rescue is sufficient to surpass the therapeutic threshold required to disrupt or slow down FTLD-*GRN* pathogenesis.

## Conclusions

In summary, we show that gentamicin G418 increases PGRN through PTC readthrough in cellular models of FTLD due to a pathogenic progranulin nonsense mutation. The enhancer compound CDX5–288 potentiates the readthrough effects of G418 in vitro and increases PGRN over G418 treatment alone. We further show that increased progranulin through PTC readthrough ameliorates lysosomal dysfunction in R493X^−/−^ KI neurons, showing a functional impact of raised PGRN. Intracerebroventricular administration of G418 in mice expressing the tagged human R493X nonsense mutation achieves in vivo PTC readthrough, and a single drug injection increases whole brain PGRN levels. Taken together, our findings suggest that PTC readthrough may be a potential therapeutic strategy for a subset of patients with FTLD bearing a *GRN* nonsense mutation.

## Supplementary information

**Additional file 1.** Supplemental figures and tables.

## Data Availability

All data generated and analyzed during the current study are included in this published article and its supplementary files.

## Abbreviations

AAV: Adenoassociated virus
CTSD: Cathepsin D
FTLD: Frontotemporal lobar degeneration
FTLD-*GRN*: Frontotemporal lobar degeneration due to a *GRN* mutation
*GRN*: Progranulin gene
hiPSC: Human induced pluripotent stem cell
ICV: Intracerebroventricular
NCL: Neuronal ceroid lipofuscinosis
NMD: Nonsense mediated mRNA decay
NPC: Neural progenitor cell
PGRN: Progranulin protein
PTC: Premature Termination Codon
