# Supplementary material for: Premature termination codon readthrough upregulates progranulin expression and improves lysosomal function in preclinical models of GRN deficiency
Source: Mol Neurodegener. 2020 Mar 16;15:21. doi: 10.1186/s13024-020-00369-5 (PMC7075020; doi:10.1186/s13024-020-00369-5)
Supplement: Supplementary file 1 — Additional file 1. Supplemental figures and tables. [file 13024_2020_369_MOESM1_ESM.docx]

**Supplemental Figures and Tables**

**
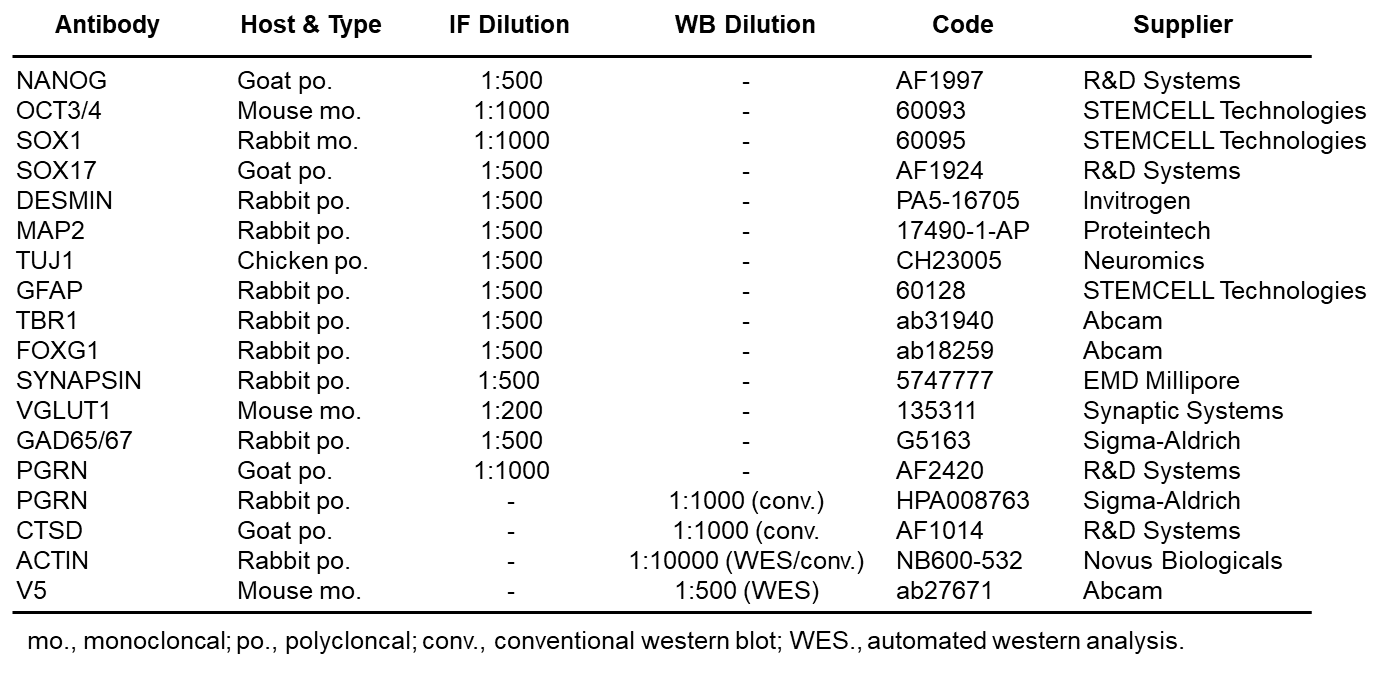

Table S1.


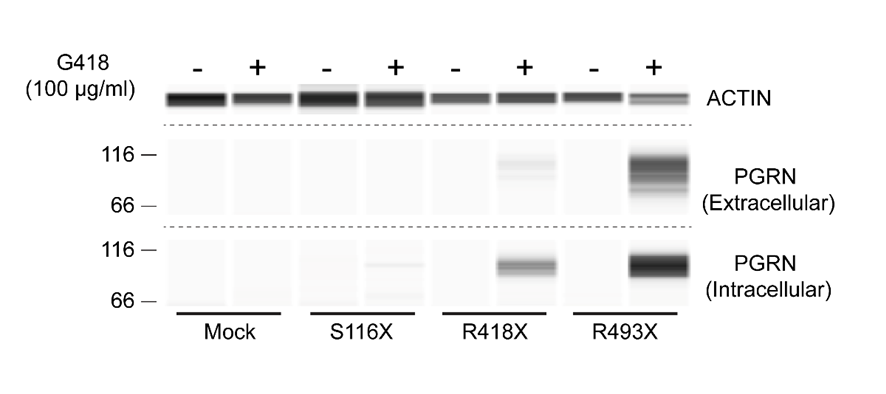
Fig. S1.** HEK293 cells transiently transfected with *GRN-V5* expression constructs bearing the indicated nonsense mutations were treated with G418 for 72 h. Cell culture supernatants (extracellular) and cell lysates (intracellular) were subjected to automated capillary electrophoresis western analysis. Full-length PGRN was detected with a V5 antibody. Actin was measured in cell lysates as a loading control. The proportion loaded was 15-20 fold lower for the extracellular samples than for the intracellular samples.

**
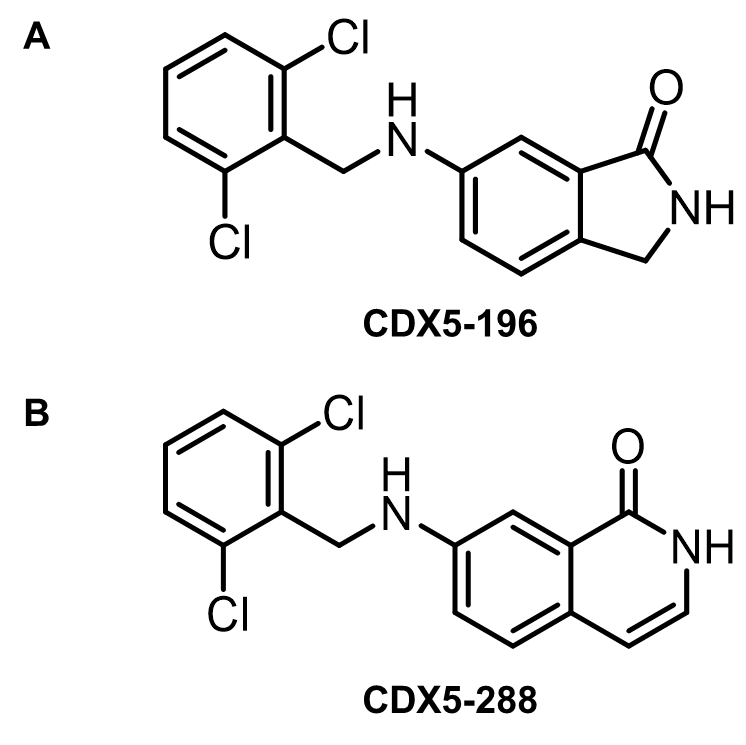
**

**Fig. S2.** Stru­cture of CDX5-196 (**a**) and CDX5-288 (**b**).

**
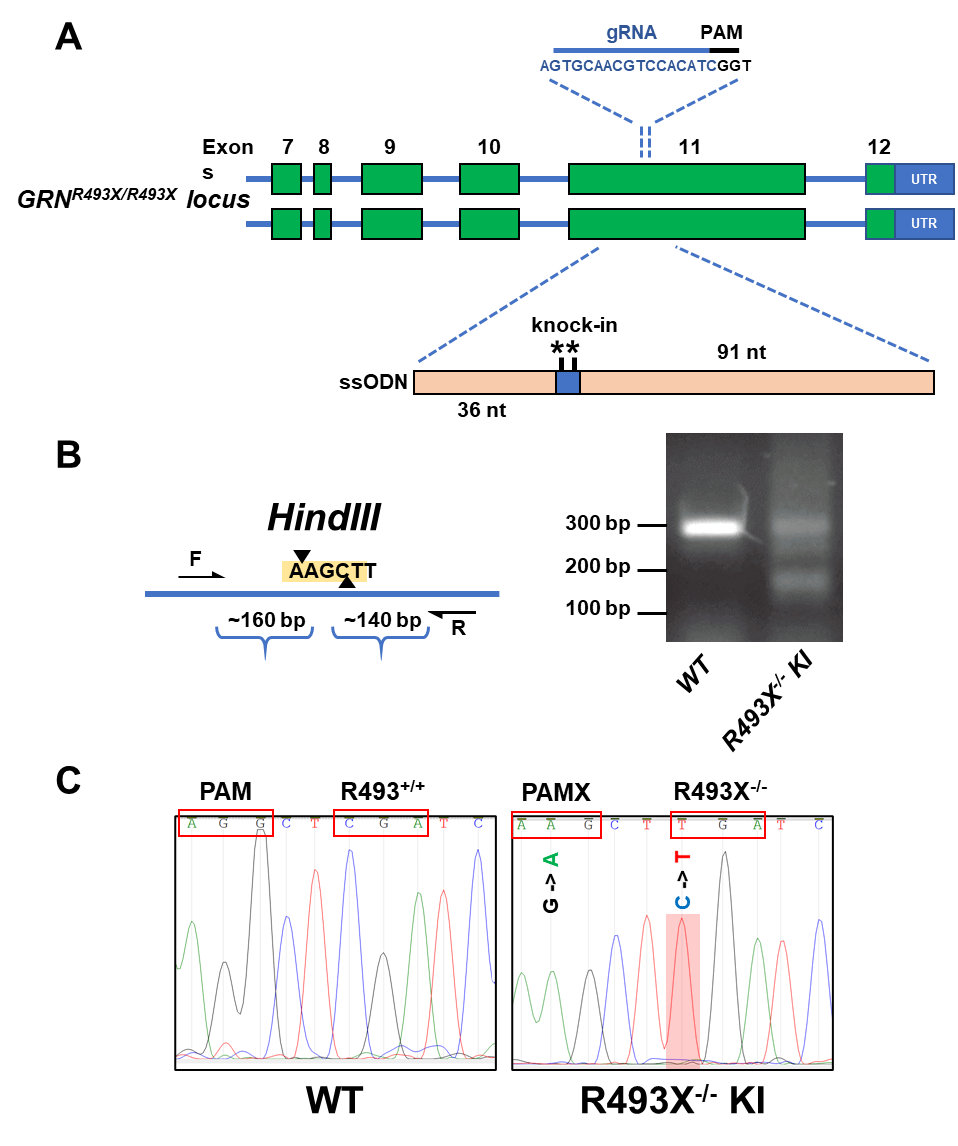
**

**Fig S3. Generation of isogenic CRISPR-gene knock-in R493X^-/-^ hiPSC line from WT clone. a**, Schematic representation of the gene-editing strategy using the CRISPR/Cas9 system. **b**, Clonal screening for the silent introduction of the AAGCTT HindIII restriction enzyme site to identify potential R493X knock-in clones. **c**, Simultaneous Sanger sequencing of the R493 codon region in both *GRN* alleles in WT and isogenic R493X^-/-^ KI clone confirming homozygous introduction of the TGA nonsense codon at codon 493 (red highlight) and silent deletion of the PAM site.


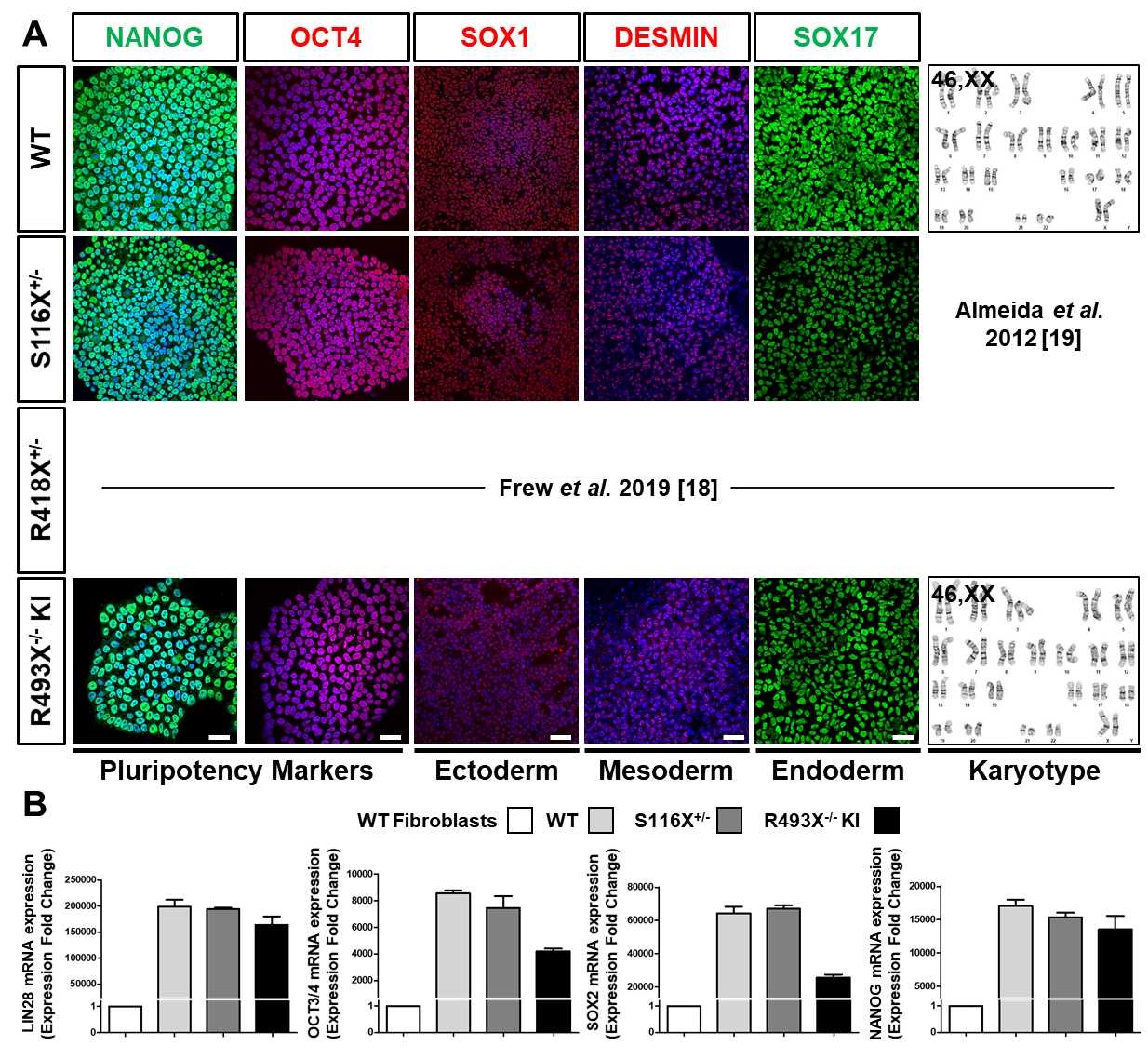


**Fig. S4.** Generation and characterization of FTD-*GRN* patient-specific hiPSCs. **a**, Immunofluorescence analysis of pluripotent markers in WT, S116X^+/-^, R418X^+/-^, and R493X^-/-^ KI hiPSC lines, and their respective normal karyotypes. In vitro trilineage differentiation of WT, S116X^+/-^, R418X^+/-^, and R493X^-/-^ KI hiPSC lines, cells were immunostained with SOX1 (ectoderm), desmin (mesoderm), SOX17 (endoderm). Cell nuclei were counterstained with DAPI (blue) except for SOX17. Scale bar, 50 µm. **b**, mRNA expression of pluripotent reprogramming factors in WT, S116X^+/-^, R418X^+/-^, and R493X^-/-^ KI hiPSC lines relative to the values in WT fibroblasts, as assessed by qPCR. Values are shown as mean ± SEM.

**
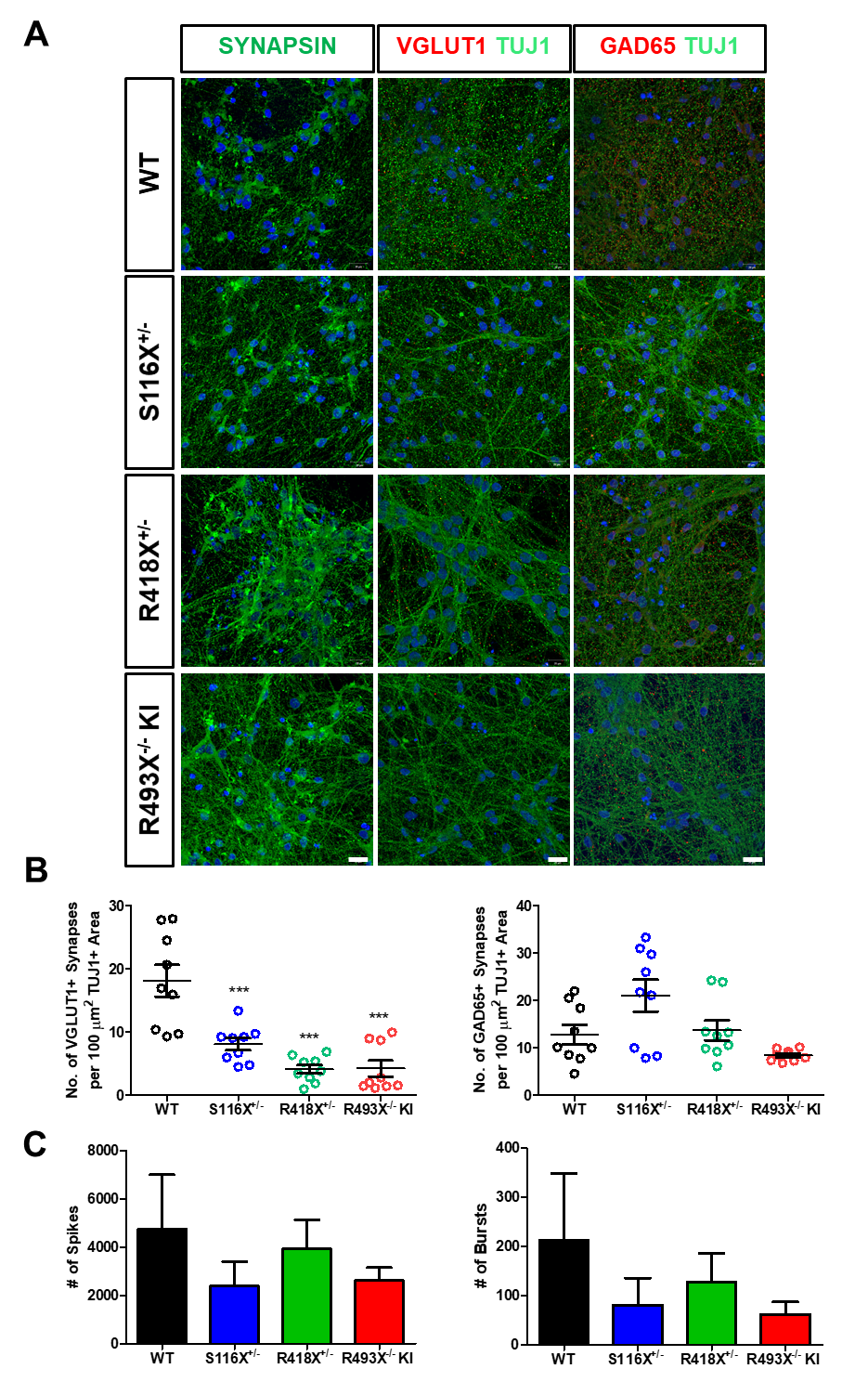
**

**Fig. S5.** Cortical neuron cultures form synaptic connections as they develop neural networks as indicated by the immunofluorescent detection of synaptic protein markers. **a**, Representative immunofluorescence images of DIV 50 WT, S116X^+/-^, R418X^+/-^, and R493X^-/-^ KI hiPSC-derived cortical neurons stained for synapsin, VGLUT1 (excitatory), and GAD65 (inhibitory). Cell nuclei were counterstained with DAPI (blue). Scale bar, 20 µm. **b**, Quantification of the synaptic density (excitatory/inhibitory) per 100 µm^2^ area of TUJ1^+^ staining in DIV 50 WT, S116X^+/-^, R418X^+/-^, and R493X^-/-^ KI hiPSC-derived cortical neuron cultures. n = 3 independent cultures, 3 images per biological replicate; values are shown as mean ± SEM; *** p < 0.0001 was determined by one-way ANOVA with Tukey’s multiple comparison test. **c**, Electrophysiological properties of DIV 50 WT, S116X^+/-^, R418X^+/-^, and R493X^-/-^ KI hiPSC-derived cortical neurons co-cultured with WT human astrocytes were recorded via multielectrode array to quantify the number of spikes and bursts detected over a 10 min interval. Values are shown as mean ± SEM.


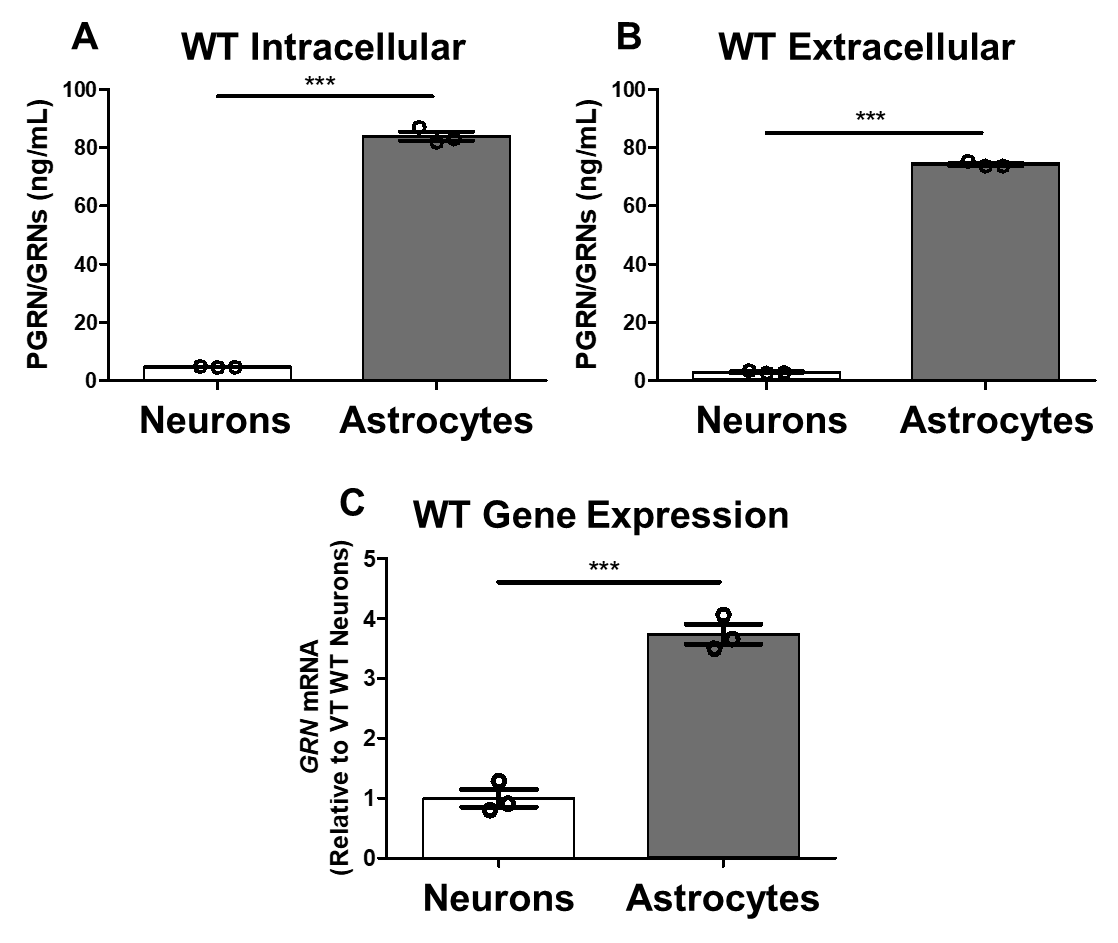


**Fig. S6.** Progranulin expression is significantly greater in the intra- (**a**) and extracellular (**b**) fractions of WT hiPSC-derived astrocytes compared to cortical neurons. WT and R493X^-/-^ KI hiPSC-derived cortical neurons and astrocytes were cultured in fresh medium for 72 h. Intracellular (1 mg/mL lysate) and extracellular (25X concentrated supernatant) samples were subjected to PGRN ELISA. qPCR analysis of human *GRN* mRNA levels in vehicle-treated (VT) DIV 50 WT hiPSC-derived cortical neurons and DIV 60+ WT hiPSC-derived astrocytes (**c**). Relative *GRN* mRNA levels were normalized to the mean of *GAPDH* and *HPRT1* housekeeping genes and compared to VT neurons. n = 3 independent cultures; values are shown as mean ± SEM; *** p < 0.0001 was determined by Student’s *t*-test.


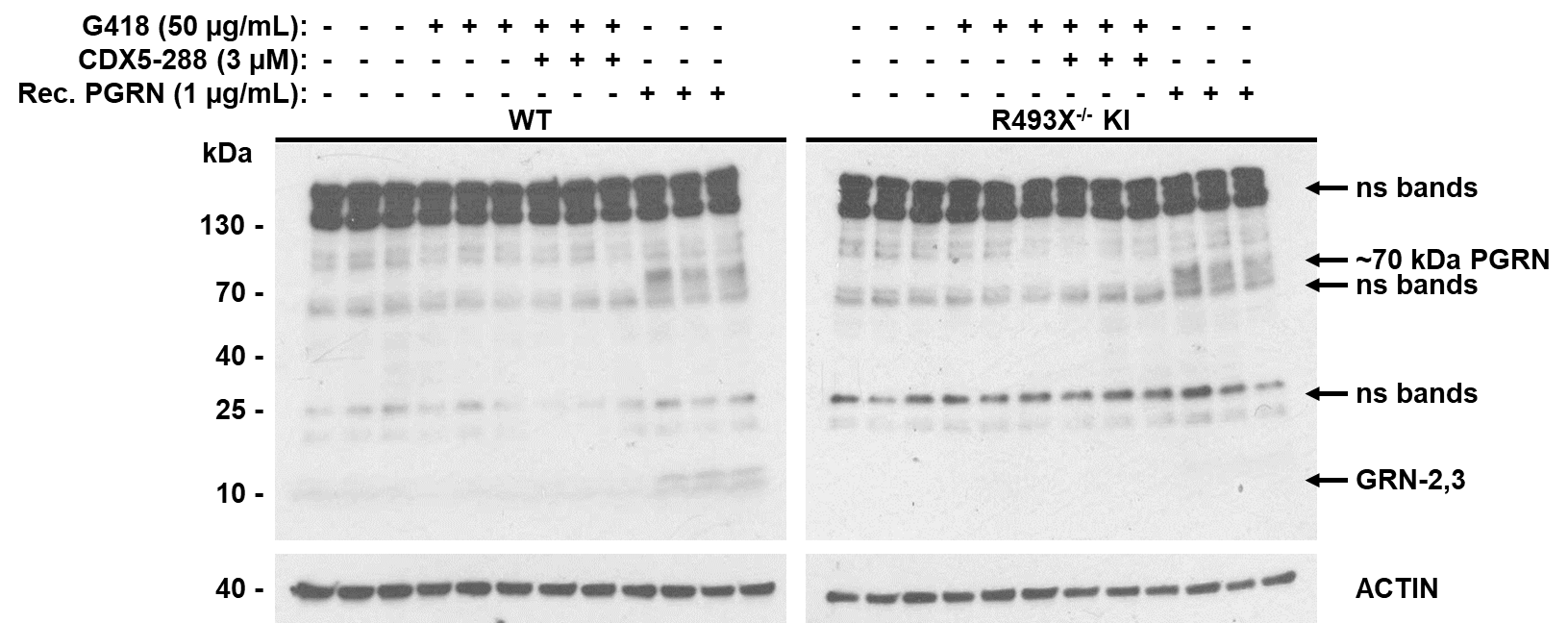


**Fig. S7.** Detecting PGRN in DIV 80 WT and R493X^-/-^ KI hiPSC-derived cortical neuron cultures treated with G418, combination with CDX5-288, or rec. human PGRN. Expression of intracellular ~70 kDa PGRN was only detected in WT and R493X^-/-^ KI neurons treated with 1 µg/mL rec. human PGRN. GRN-2,3 peptides were only detected in WT neurons treated with rec. human PGRN. Neuronal lysates were analyzed by Western blotting, using actin as the loading control. ns = nonspecific.

**
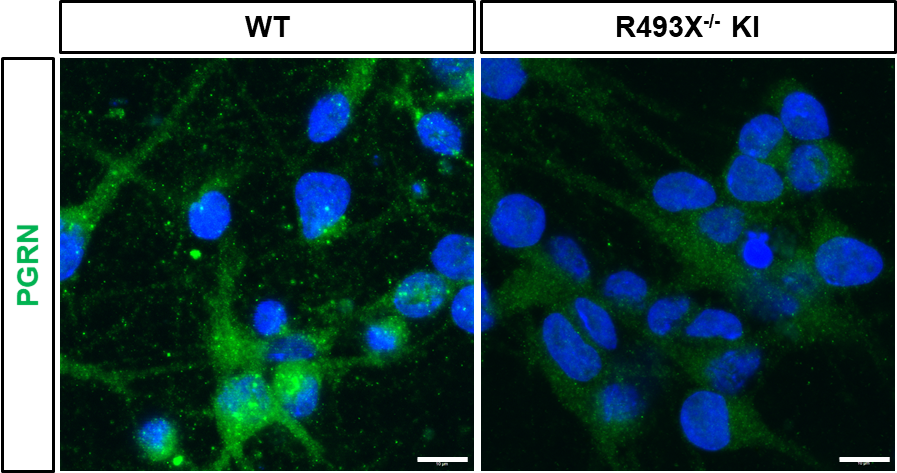
**

**Fig. S8.** CRISPR isogenic homozygous knock-in (R493X^-/-^ KI) nonsense mutant hiPSC-derived cortical neurons (DIV 50) possess greatly reduced and more diffuse PGRN expression than the WT parental line, as detected by immunofluorescence. Cell nuclei were counterstained with DAPI (blue). Scale bar, 10 µm.

**Fig. S9.** Quantification of G418 cytotoxicity in R418X^+/-^ iPSC-derived neurons treated with escalating doses of G418. Neurons were treated for 120 h with fresh media/drug solutions replaced after 72 h. Cultures were stained with Hoechst dye and counted using a Cellomics ArrayScan^TM^ device. n = 3 independent cultures, 12 images per biological replicate; values are shown as mean ± SEM.

**
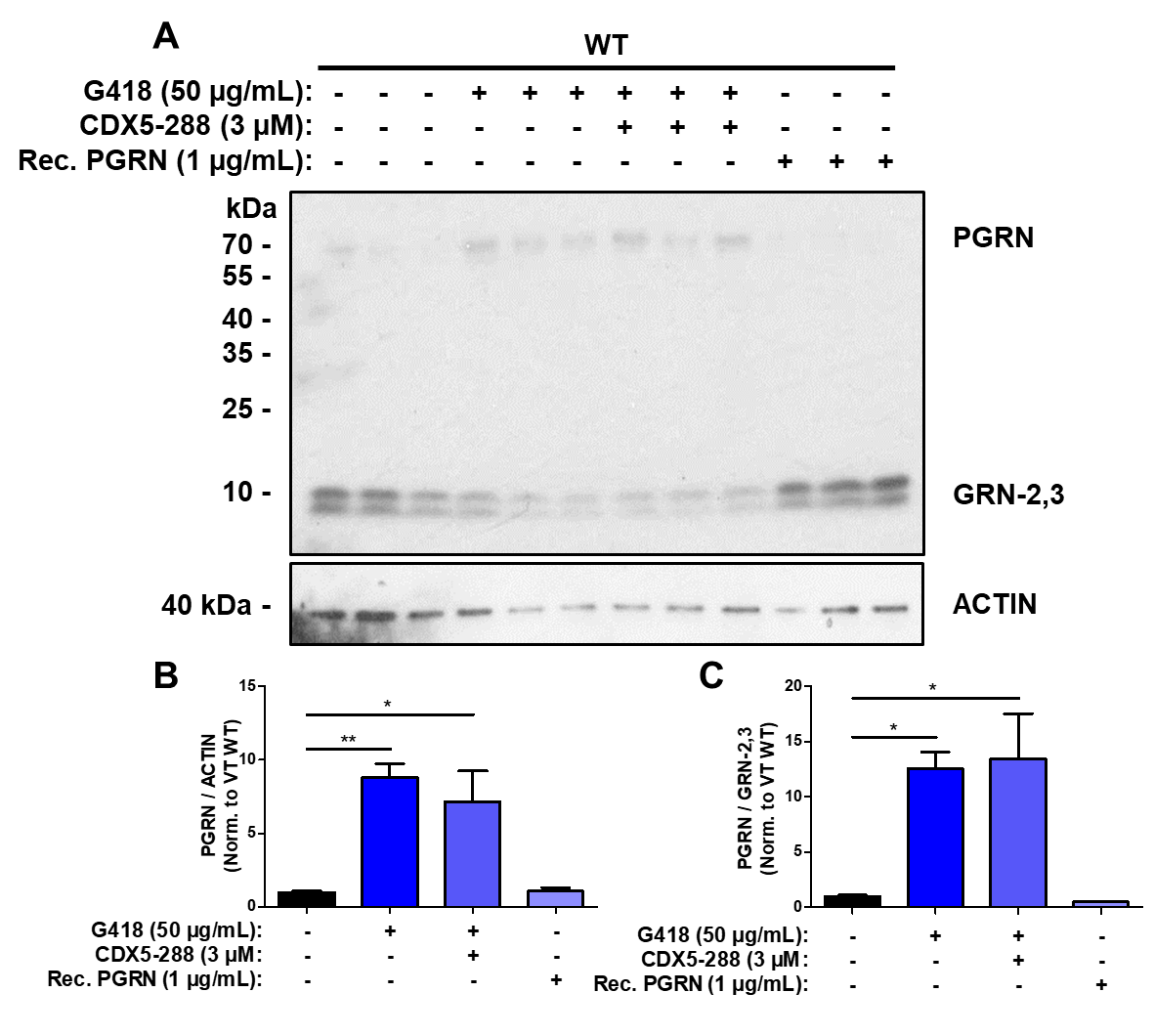
**

**Fig. S10.** G418 disrupts PGRN homeostasis in WT hiPSC-derived DIV 60+ astrocyte cultures. Expression of intracellular full-length PGRN and GRN-2,3 peptides in G418, combination, and rec. human PGRN treated WT astrocyte lysates analyzed by Western blotting (***A***), using actin as the loading control. Densitometric quantification PGRN and the ratio of PGRN:GRN-2,3 peptide expression in the aforementioned astrocyte lysates normalized to vehicle-treated WT levels. n = 3 independent cultures; values are shown as mean ± SEM; * p < 0.05, ** p < 0.01 was determined by one-way ANOVA with Tukey’s multiple comparison test.

**
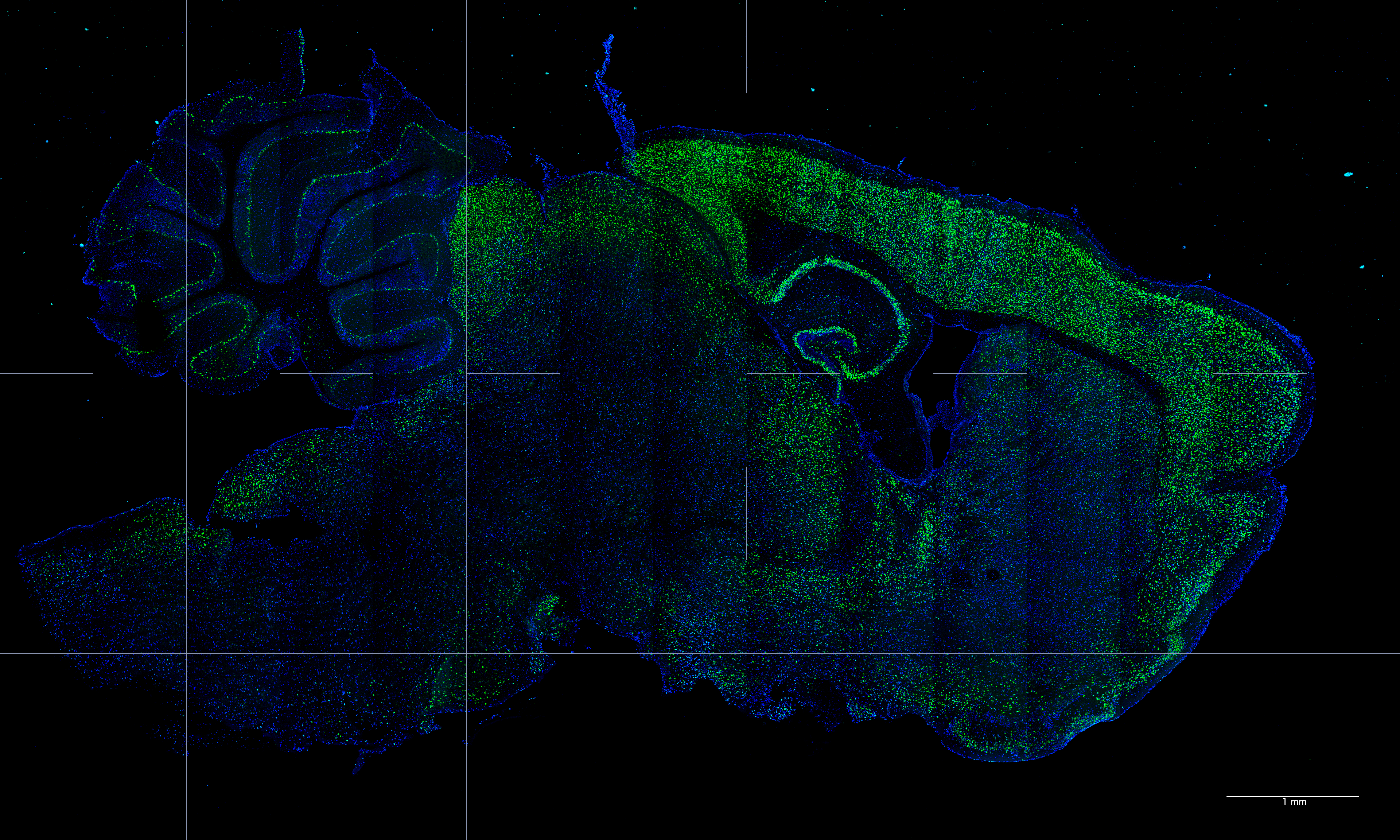
**
**Fig. S11.** Demonstration of P0 ICV injection technique using an AAV9-eGFP-Cre vector with expression driven by the human *SYN1* promoter. Diffuse brain-wide eGFP (green) expression 2 months (~10 weeks old) following bilateral P0 ICV injection. Cell nuclei were counterstained with DAPI (blue). Scale bar, 1 mm.
